# Safety of Eplerenone for Kidney-Transplant Recipients with Impaired Renal Function and Receiving Cyclosporine A

**DOI:** 10.1371/journal.pone.0153635

**Published:** 2016-04-18

**Authors:** Jean-Philippe Bertocchio, Coralie Barbe, Sylvie Lavaud, Olivier Toupance, Pierre Nazeyrollas, Frederic Jaisser, Philippe Rieu

**Affiliations:** 1 Nephrology, Dialysis and Transplantation Unit, Reims University Hospital, Avenue Cognacq Jay, 51092 Reims CEDEX, France; 2 Biostatistics and methodology unit, Reims University Hospital, Avenue Cognacq Jay, 51092 Reims CEDEX, France; 3 INSERM UMRS 1138 –Team 1, Research Centre of Cordeliers, 15 rue de l’école de médecine, 75006 Paris CEDEX, France; University Jean MONNET of SAINT-ETIENNE, UNITED STATES

## Abstract

**Background:**

Animal studies have highlighted the role of vascular mineralocorticoid receptor during Cyclosporine A-induced nephrotoxicity. Mineralocorticoid receptor antagonists could improve kidney survival but are not commonly used during renal impairment and in association with several immunosuppressive drugs due to a supposed higher risk of adverse events. We tested the tolerance of eplerenone according to its expected adverse events: hyperkalemia, metabolic acidosis, hypotension, acute kidney failure, or any other adverse event.

**Methods:**

We conducted a single-center, prospective, open-label study in 31 kidney-transplant recipients with impaired renal function (30 and 50 mL/min/1.73m^2^) and receiving cyclosporine A. All patients received eplerenone 25 mg/d for 8 weeks. Serum potassium, renal function and expected adverse events were closely monitored.

**Results:**

Eight patients experienced mild hyperkalemia (>5 mmol/L), one moderate hyperkalemia (>5.5 mmol/L) and had to receive potassium-exchange resin. No severe hyperkalemia (>6 mmol/L) occurred. One acute kidney failure was observed, secondary to diarrhea. Basal serum potassium and bicarbonate were independently associated with a higher risk of developing mild hyperkalemia (>5 mmol/L) under treatment (OR 6.5, *p* = 0.003 and 0.7, *p* = 0.007, respectively). A cut-off value of 4.35 mmol/L for basal serum potassium was the best factor to predict the risk of developing mild hyperkalemia (>5 mmol/L).

**Conclusions:**

Until eGFR falls to 30 mL/min/1.73m^2^, eplerenone could be safely given to kidney-transplant recipients receiving cyclosporine A, if kalemia is closely monitored. When renal function is impaired and if basal kalemia is >4.35 mmol/L, then clinicians should properly balance risk and benefit of eplerenone use and offer dietary advice. An adequately powered prospective randomized study is now needed to test its efficiency (and safety) in this population.

**Trial Registration:**

ClinicalTrials.gov NCT01834768

## Introduction

Calcineurin inhibitors (CNIs), such as Cyclosporine A (CsA) or tacrolimus, are the most commonly used maintenance immunosuppressive drugs after kidney transplantation [1] even if CNIs could lead to nephrotoxicity [2]. The mechanisms underlying CsA-induced nephrotoxicity (CIN) remain not fully elucidated [3]. Renal hemodynamic plays a central role during acute CIN: renal vasoconstriction has been reported as an initial event linked to CIN [3]. CsA is associated with renal afferent arteriolar vasoconstriction in rats and tubular injury during acute CsA nephrotoxicity [4].

The pharmacological antagonism of Mineralocorticoid Receptor (MR) reduces both cardiovascular and all-cause morbidity and/or mortality during chronic related (or not) heart failure [5, 6]. The MR expressed in endothelium and smooth muscle cells participates to the control of vascular tone: both endothelial and vascular smooth muscle MR modulate the responses to vasodilators and vasoconstrictors [7, 8]. Pharmacological antagonism of MR by both spironolactone [9, 10] and eplerenone [11, 12] is highly efficient to blunt CIN in experimental models. The vascular smooth muscle MR has been recently shown to play a key role during acute CIN in mice by preventing increased renal vascular resistance in acute CIN [13]: this could explain, at least partially, the beneficial effects of MR antagonism in CIN.

Chronic renal impairment could limit the use of MR antagonists (MRAs): even if hyperkalemia is feared, spironolactone and eplerenone could be safely used if a close monitoring of kalemia and renal function is ensured [14, 15]. However, the higher frequency of polypharmacy in chronic kidney disease patients could lead to drug-drug interactions and limit MRAs use, especially during kidney transplantation when immunosuppressive drugs metabolized by the P450 cytochrome (like CsA) are necessary. MRAs are not commonly used in this population despite the potential benefits to reduce cardiovascular risk and CIN after renal transplantation.

Gonzalez Monte *et al*. reported the benefits of adding spironolactone to a dual-blockade renin–angiotensin–aldosterone system (by both angiotensin-converting enzyme inhibitor [ACE-I] and type-2 angiotensin-receptor blockers [ARB]) in 11 kidney-transplant recipients with persistent proteinuria: after 6 months, proteinuria had decreased significantly with no adverse event [16]. Serum potassium remained stable (no severe hyperkalemia) and no serum bicarbonate was reported [16]. Since MRAs have never been tested in CsA-treated kidney-transplant recipients with impaired renal function, the present study was designed to test the tolerance of eplerenone in this population.

## Subjects and Methods

We conducted a single-center, prospective, open-label study. The primary endpoint was the tolerance to eplerenone, assessed by the occurrence of the following expected adverse events: severe hyperkalemia (>6 mmol/L), metabolic acidosis (serum bicarbonate <15 mmol/L), hypotension (systolic blood pressure <100 mmHg), acute kidney failure (increase in serum creatinine >30% from baseline), or any adverse event that required discontinuation of eplerenone. Eplerenone was chosen due to its lower affinity for other steroid (progesterone, androgen and glucocorticoid) receptors and the absence of long-acting metabolites: this could lead to less frequent adverse events. We calculated the number of patients to include based upon the risk of severe hyperkalemia (>6 mmol/L), which was considered as the major adverse event.

Levels of kalemia were defined during the study as follow: normal (3.5 to ≤5 mmol/L), mild hyperkalemia (>5 to 5.4 mmol/L), moderate hyperkalemia (>5.5 to 5.9 mmol/L) and severe hyperkalemia (>6 mmol/L).

### Study design

**Fig 1:**
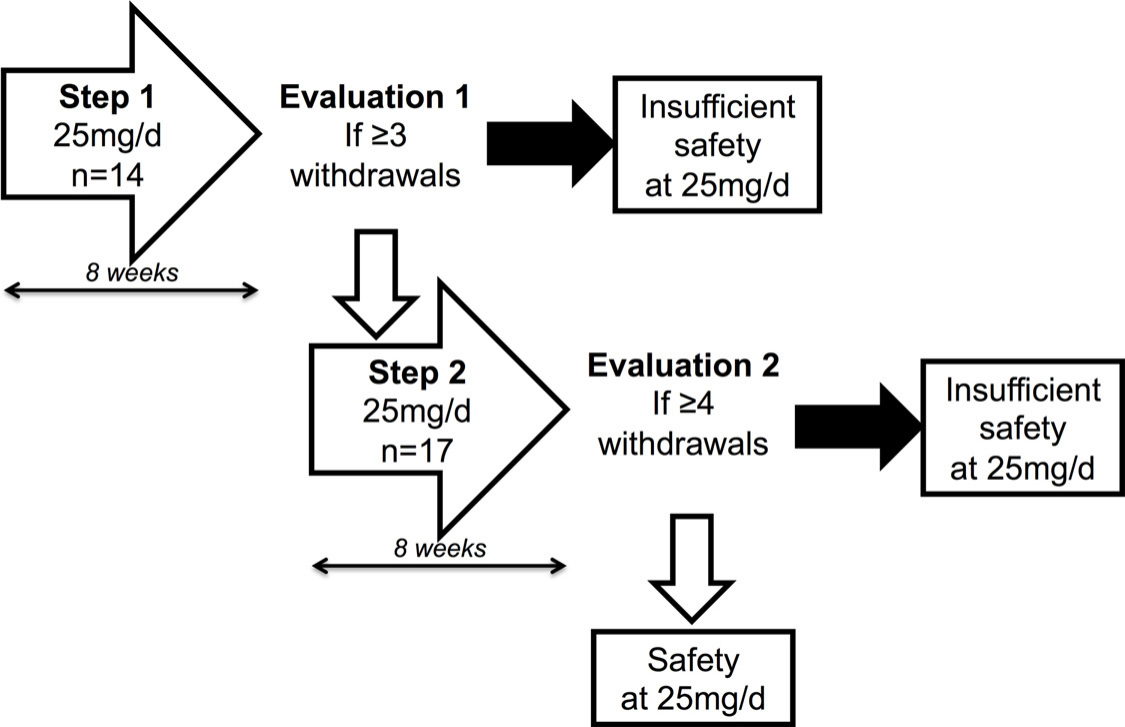
Design of the EpleCsAT: Safety trial. Sequential inclusion was performed: 14 patients during step 1; then 17 new patients during step 2.

We performed the study by using a two-steps Simon’s plan (**Fig 1**) [17]. During the first step, 14 patients took eplerenone 25 mg/d for 8 weeks. This posology was chosen to be the minimum efficient. If three or more adverse events occurred, then study had to be discontinued. If not, 17 new patients were included within step 2 and also received the same treatment for 8 weeks. If four or more adverse events occurred in both steps (1 and 2), then study had to be discontinued, and the alternate hypothesis (a risk of adverse events >20%) could not be rejected: i.e., the safety of eplerenone could not be concluded. If not, we could conclude that eplerenone at 25 mg/d could be safety used in such a population.

All included patients were aged >18 years on the date of inclusion, belonged to a healthcare system, gave their informed written consent, had a functional kidney allograft for at least 1 year from the date of inclusion, was receiving CsA, and had impaired renal function, estimated by the MDRD formula [18], at between 30 and 50 mL/min/1.73 m^2^. Exclusion criteria were serum potassium of ≥5 mmol/L on the date of inclusion; one or more incidences of severe hyperkalemia (≥6 mmol/L), for whatever reason; currently receiving potassium-exchange resin treatment; on-going pregnancy or lack of effective contraception during the whole study period; uncontrolled high arterial blood pressure (systolic blood pressure >140 mmHg); orthostatic hypotension; systolic arterial blood pressure ≤110 mmHg; heart failure within the 3 months before the date of inclusion or chronic heart failure (NYHA III or IV); severe hepatic failure (Child-Pugh C score); allergy to one or more of the components of eplerenone (INSPRA^®^); on-going treatment, including spironolactone or eplerenone; on-going treatment that could not be withdrawn during the study period: e.g., potassium-sparing diuretics, potassium salts, CYP3A4 enzyme inhibitors other than CsA; malabsorption syndrome; abnormal galactose metabolism or a deficiency of galactase; on-going non-steroidal anti-inflammatory treatment, or lithium, or another nephrotoxic agent; or on-going treatment with a double-blockade of the renin–angiotensin–aldosterone system with ACE-I and ARB. The treatment could include ACE-I or ARB, but not in combination.

Clinical parameters (body weight, blood pressure, and adverse events) were monitored on days (D) 0, 14, 28, and 56. Serum potassium was closely monitored on days 0, 2, 7, 14, 21, 28, 35, 42, 49, and 56. Other biological parameters (such as serum creatinine and bicarbonate) were monitored on D 0, 14, 28, and 56.

At any time during the study period, adverse events that required discontinuation of eplerenone included serum potassium >6 mmol/L, serum potassium >5.5 mmol/L under potassium-exchange resin, metabolic acidosis assessed by a serum bicarbonate <15 mmol/L, and any other clinical outcome that required discontinuation of eplerenone.

### Statistical methods and analyses

According to previously reported data, the probabilities to develop hyperkalemia (> 6 mmol/L) during 8 weeks of eplerenone treatment are <7% with the 25mg/d dose and <10% with the 50mg/d dose [5, 6, 16, 19]. Included patients exerted a better renal function than the population of the present study: herein, estimated glomerular filtration rate (eGFR) range was fixed between 50 and 70 mL/min/1.73m^2^. In our population, the expected risk (null hypothesis, H_0_) was supposed to be < 7% whereas a risk higher than 20% of developing major hyperkalemia (>6 mmol/L) was considered unacceptable (alternative hypothesis, Ha). Using a sample proportion test, the power to detect this adverse outcome was calculated at 95% (the β risk was 5%). Otherwise, in such conditions, the risk of not identifying an unacceptable risk of major hyperkalemia (>6 mmol/L) under eplerenone in these patients is 5%: the α risk was calculated at 22.5%. With these hypotheses, the inclusion of 31 patients was required: if 4 (/31) or more patients had to stop the treatment, the safety (< 20%) could not be assumed.

Quantitative data are described by their median and range and qualitative data as numbers and percentages. Variations of serum potassium at different times were evaluated using variance analysis for repeated measures. Comparisons between patients with mild hyperkalemia (>5 mmol/L) during the study protocol and those with normal kalemia (<5 mmol/L) at anytime of the study period were performed using univariate analyses (Wilcoxon's test or Fisher’s exact test, as appropriate) and multivariate analyses (stepwise logistic regression). The multivariate stepwise logistic regression included only significant factors at *p* ≤ 0.10 with entry and removal limits set at 0.10: basal cyclosporine A posology, creatininemia, serum potassium and bicarbonate. Sensitivities and specificities of basal serum potassium and bicarbonate were calculated, and a receiver-operating characteristic (ROC) curve was calculated to determine a cut-off value with optimal sensitivity and specificity: the statistical software (SAS) calculated automatically the coordinates of the ROC curve and calculated both the sensitivity and specificity (1—specificity for more precision) for all coordinates. Then, the cut-off value obtaining the best ratio between the sensitivity and the specificity was chosen.

Whatever the test used, a *p*-value <0.05 was considered statistically significant. All statistical analyses were performed using SAS software, release 9.3 (SAS INC, Cary, California).

### Ethical considerations

This trial (**S1 and S2 Figs**) received specific agreements from an appropriate independent ethics committee, was registered in the European registry (EudraCT 2011-003759-20) and in clinicaltrials.gov (NCT01834768) and has therefore been performed in accordance with the ethical standards laid down in an appropriate version of the Helsinki Declaration of 1975, as revised in 2000, as well as the Declaration of Istanbul 2008. All persons gave their informed written consent prior to their inclusion to the study. The clinical and research activities being reported are consistent with the Principles of the Declaration of Istanbul as outlined in the “Declaration of Istanbul on Organ Trafficking and Transplant Tourism”.

## Results

**Fig 2:**
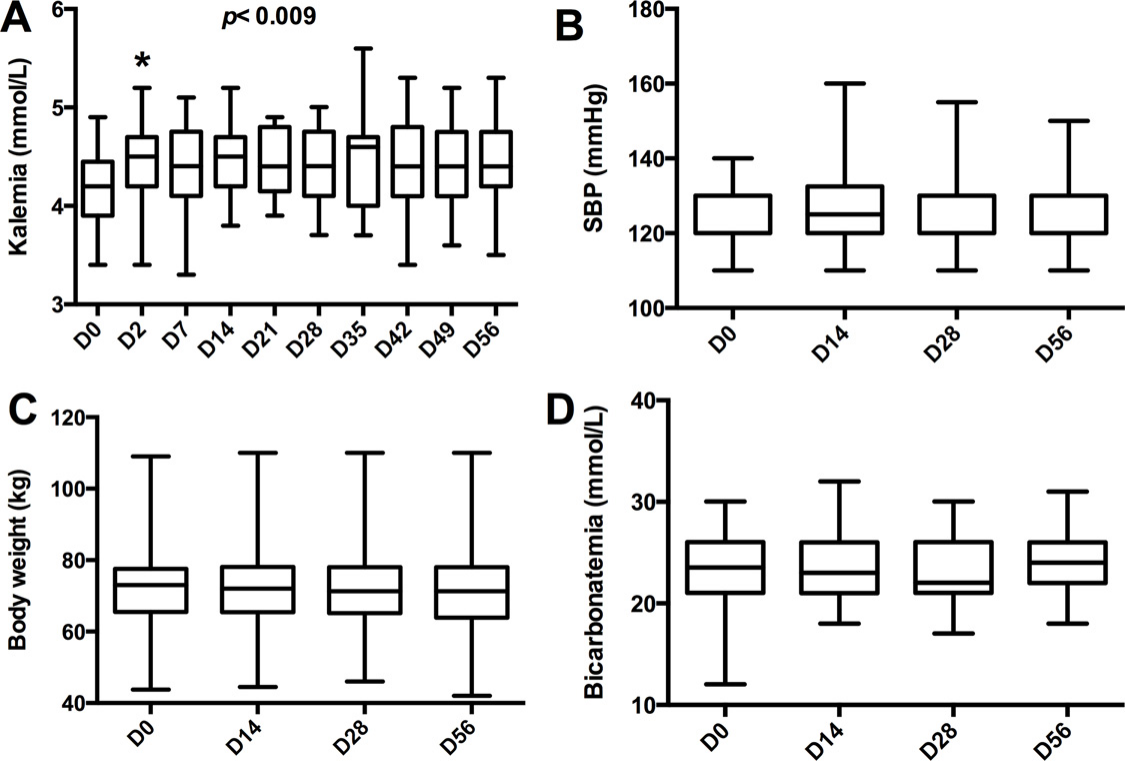
Eplerenone induced mild hyperkalemia. **(A)** Kalemia increased from day 2 (D2) and became stable during the treatment period. **(B)** Systolic blood pressure (SBP), **(C)** body weight, and **(D)** serum bicarbonate did not change during the treatment period. Data are represented as their median and range (whiskers). * *p* <0.05 *vs*. D0.

A total of 31 patients were included (**Table 1**) and all completed the study period (8 weeks), except one (last follow-up on D35 due to an unplanned move). Serum potassium increased slightly from baseline (4.2±0.4 mmol/L): on d2, serum potassium became increased and then remained in a steady state (**Fig 2A**). Nine patients experienced at least one episode of mild hyperkalemia (>5 mmol/L) but there was only one episode of moderate hyperkalemia (>5.5 mmol/L). This patient received a specific intervention (potassium-exchange resin) on D35. Half the incidences of mild hyperkalemia (>5 mmol/L) occurred within 7 days after beginning eplerenone treatment.

**Table 1:** Characteristics of included patients.

***Demography***	*n* = 31
Age (years)	56 [32–70]
Gender ratio (M/F)	18/13
Time since transplantation (months)	126 [18–326]
Body-mass index at inclusion (kg/m^2^)	23.8 [18.2–36.8]
Diabetes, *n* (%)	3 (10)
***Biology***	
Creatininemia (μmol/L)	145 [87–239]
eGFR (mL/min/1.73 m^2^)	41 [26–59]
Serum potassium at inclusion (mmol/L)	4.2 [3.4–4.9]
Serum bicarbonate at inclusion (mmol/L)	24 [12–30]
Natriuresis at inclusion (mmol/d)	136 [29–360]
Kaliuresis at inclusion (mmol/d)	60 [0–176]
Proteinuria at inclusion (mg/d)	123 [0–648]
***Drug therapies***	
Cyclosporine posology at inclusion (mg/kg/d)	2.1 [1.4–4.0]
Cyclosporinemia at inclusion (ng/mL)	94 [38–152]
MMF/azathioprine, *n* (%) / *n* (%)	24 (77) / 4 (1)
ACE-i/ARB, *n* (%) / *n* (%)	13 (42) / 6 (2)
Diuretics, *n* (%)	12 (39)
β-blockers, *n* (%)	14 (45)
Oral bicarbonate, *n* (%)	7 (23)
Steroids, *n* (%)	5 (16)

M: male; F: female; eGFR: estimated glomerular-filtration rate; MMF: mycophenolate mofetil. Data are expressed by their median [range].

Three patients presented with other adverse events: two unspecific outcomes (diarrhea and sweats) and one acute kidney injury (>30% increased creatininemia from baseline) on D56, secondary to acute diarrhea. None of these adverse events needed specific management. We observed no modifications to systolic blood pressure (**Fig 2B**), body weight (**Fig 2C**), or serum bicarbonate (**Fig 2D**). Other biological or clinical parameters remained stable.

The risk of at least one episode of mild hyperkalemia (>5 mmol/L) under eplerenone was studied using baseline data: demographic and biological parameters were analyzed as well as treatments. Two groups were individualized (patients with at least one episode of mild hyperkalemia (>5 mmol/L) *versus* others). After stepwise multivariate analyses (including CsA posology, creatininemia, serum potassium and bicarbonate), only serum potassium and bicarbonate at baseline were independently associated with a higher risk of developing at least one episode of mild hyperkalemia (>5 mmol/L) under eplerenone treatment (**Table 2**). Higher serum potassium at baseline was associated with a higher risk (OR 6.5 [1.4;30.5]) of developing mild hyperkalemia and lower serum bicarbonate was also associated with a higher risk (OR 0.7 [0.5;0.9]) of developing mild hyperkalemia.

**Table 2:** Candidate parameters for predicting the risk of mild hyperkalemia.

Parameter	Kalemia >5 mmol/L (*n* = 9)	No kalemia > 5 mmol/L (*n* = 22)	Univariate analysis^#^	Multivariate analysis*		
			*p*	*p*	OR	95%CI
***Demography***						
Age (years)	50.7 [32.7–70.1]	57.4 [35.8–66.5]	0.31			
Gender (M/F)	7/2	11/11	0.12			
Body weight (kg)	75.0 [66–90]	70.1 [43.8–109]	0.17			
Body-mass index (kg/m^2^)	23.8 [23.0–28.2]	24.4 [18.2–36.8]	0.84			
Diabetes at inclusion (*n*)	0	3	0.34			
Time since transplantation (months)	152.0 [24–326]	119.5 [18–264]	0.29			
Systolic blood pressure (mmHg)	120 [110–140]	130 [110–140]	0.42			
***Biology***						
Creatininemia on day 0 (μmol/L)	170.0 [121.0–232.0]	138.0 [87.0–239.0]	0.06			
eGFR (mL/min/1.73 m^2^)	36.0 [26.0–53.0]	44.5 [26.0–59.0]	0.17			
Serum potassium at baseline (mmol/L)	4.7 [4.0–4.9]	4.1 [3.4–4.7]	***<0*.*01***	***0*.*003***	6.5	[1.4;30.5]
Kaliuresis (mmol/d)	62.0 [33.0–92.0]	57.0 [0–176]	0.33			
Kaliuresis/creatininuria (mmol/mmol)	4.8 [0.3–7.0]	4.7 [0–21.5]	0.57			
Natriuresis (mmol/d)	143 [71–300]	135.5 [29–360]	0.84			
Natriuresis/creatininuria (mmol/mmol)	10 [1.2–14.8]	12.7 [1.8–64.6]	0.25			
Serum bicarbonate at baseline (mmol/L)	21.0 [12.0–25.0]	24.0 [19.0–30.0]	***0*.*02***	***0*.*007***	0.7	[0.5;0.9]
***Drug therapy***						
Cyclosporine A posology at inclusion (mg/d)	180 [120–220]	140 [100–280]	0.08			
Cyclosporine A posology at inclusion (mg/kg/d)	2.1 [1.6–2.8]	2.0 [1.4–4.0]	0.37			
Cyclosporinemia at inclusion (ng/mL)	98 [38–145]	92.5 [40–152]	0.81			
ACE-I at inclusion (*n*)	3	10	0.26			
ARB at inclusion (*n*)	2	4	0.36			
Diuretics at inclusion (*n*)	4	8	0.29			
β-blockers at inclusion (*n*)	4	10	0.31			
Oral bicarbonate at inclusion (*n*)	2	4	0.36			
Steroids at inclusion (*n*)	1	4	0.39			

M: male; F: female; eGFR: estimated glomerular-filtration rate; OR: odds ratio; CI: confidence interval. Data are expressed as their median [range]. All urine tests were performed on 24-h urine collections.

^#^ Univariate analyses using Wilcoxon tests for quantitative variables and Fisher exact test for qualitative variables.

* Multivariate analysis by stepwise logistic regression was performed including creatininemia, serum potassium, serum bicarbonate and cyclosporine A posology on day 0.

**Fig 3:**
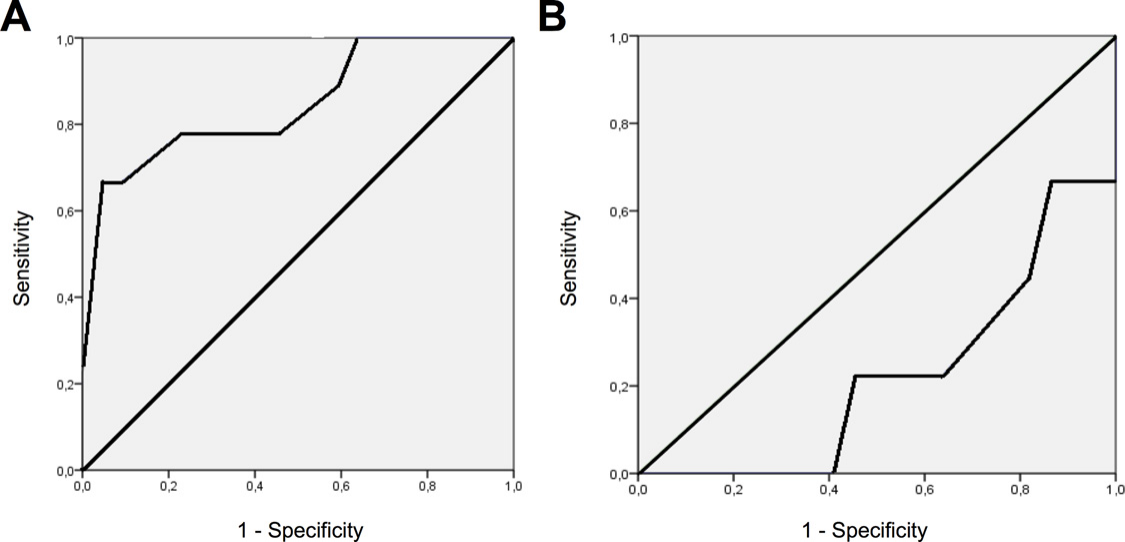
Risk factors for developing mild hyperkalemia under treatment. Receiver-operating characteristic (ROC) curves for **(A)** serum potassium and **(B)** serum bicarbonate at baseline.

ROC analyses were performed to test if a cut-off value for serum potassium and/or bicarbonate at baseline could distinguish which patients had a higher risk of developing mild hyperkalemia (>5 mmol/L) under eplerenone treatment. Only serum potassium at baseline (**Fig 3A**) showed this ability (AUC = 0.846 [0.681–1.0]), whereas serum bicarbonate at baseline (**Fig 3B**) did not (AUC = 0.222 [0.048–0.397]). Serum potassium of >4.35 mmol/L at baseline was a marker for a higher risk of developing mild hyperkalemia (>5 mmol/L) during the treatment period, with a sensitivity of 78% and a specificity of 77%.

## Discussion

During this study, we found that eplerenone could be safely given to kidney-transplant recipients treated with CsA and impaired renal function. The only acute renal failure observed during eplerenone treatment was not considered to be associated to this treatment due to the diarrhea. This gastro-intestinal adverse outcome was no longer related to eplerenone, regarding to the context of known contact. Moreover, other ongoing drugs could have facilitated this event.

After the RALES study [19], MRAs were considered to be at risk of major hyperkalemia [20], especially in patients with chronic kidney disease [21]. Most of the cases of severe hyperkalemia were due to the lack of serum potassium monitoring after initiating the treatment [22]. All CNIs increase the risk of hyperkalemia [23], especially after adding a renin–angiotensin–aldosterone-system blocker, such as ACE-I or ARB [24]: the underlying mechanisms may rely on the decreased efficacy of loop diuretics (like furosemide) [25], the activation of the sodium-chloride co-transporter [26, 27], and decreased ROMK channel activity [28].

CNIs are also associated with mild renal tubular acidosis in about one-third of patients [29]. The acidosis observed during CsA-treatment [30] can worsen potassium shift from the intracellular to the extracellular compartment: distal renal tubular acidosis [31] appears to be related to Na^+^/K^+^ ATPase pump impairment [32] under CsA-treatment. MRAs could worsen metabolic acidosis, especially when it pre-exists [33]. In our study, serum bicarbonate was closely monitored: if it was associated with a higher risk of developing mild hyperkalemia (>5 mmol/L) during the treatment period, a basal cut-off value could not be proposed. The use of oral bicarbonate was allowed and was monitored due to its possible effects on preserving renal function decline [34] and counteracting the acidotic effect of MRAs. Even if patients that had at least one episode of mild hyperkalemia (>5 mmol/L) had lower serum bicarbonate at baseline, they were not more frequently treated with oral bicarbonate (2/9 patients) than other patients (4/22, *p* = 0.36). Other treatments that could interact with the metabolism of potassium were screened: the frequencies of ACE-I, ARB, diuretics and/or β-blockers were not different between both groups.

Renal impairment is a risk factor of hyperkalemia: during chronic kidney disease, renal potassium handling increases as glomerular-filtration rate decreases [35], leading to hyperkalemia because of the loss in nephron mass. In the present study (where all patients had a renal impairment), renal function assessed by the MDRD formula [18] was not associated with a higher risk of developing mild hyperkalemia (>5 mmol/L) during the treatment period. Because of the creatininemia assay used in our study (modified Jaffe’s method), the use of the CKD-EPI formula–which necessitates an enzymatic assay–was not appropriate [36]. For ranges of eGFR between 30 to 59 mL/min/1.73m^2^, the MDRD formula misclassifies 5% of patients that should be mostly in the upper eGFR group (60 to 89 mL/min/1.73m^2^) [36]: as the included patients in the present study had eGFR ranging from 30 to 50 mL/min/1.73m^2^, misclassifications may had been rare. Even if creatininemia at baseline tended to be different between patients that experienced at least one episode of mild hyperkalemia (>5 mmol/L) during the study, eGFR was not different. Moreover, multivariate analysis included creatininemia at inclusion: it did not explain the higher frequency of mild hyperkalemia (>5 mmol/L) observed in these patients.

The risk of developing moderate to severe hyperkalemia during treatment with eplerenone is less than 10% in both hypertension and heart-failure indications, depending on the definition of hyperkalemia (>5.5 mmol/L or 6 mmol/L) and drug dosage [37]: in our study, only one (1/31, about 3%) moderate hyperkalemia (>5.5 mmol/L) was observed. Laboratory monitoring (serum potassium and renal function), after initiating MRA treatment, is the best way to prevent hyperkalemia and hospitalization [38]. Even though our cohort was relatively small (*n* = 31), higher serum potassium at baseline was associated with a higher risk of developing mild hyperkalemia (>5 mmol/L) during the treatment period. In our study, mild hyperkalemia was not associated with a higher rate of 24-h kaliuresis, neither at baseline nor during the follow-up. At a steady state, kaliuresis reflects potassium intake and is not related to a higher risk of developing hyperkalemia: this is consistent with a previous study [39].

To evaluate the risk of developing mild hyperkalemia during treatment with eplerenone, a cut-off value of 4.35 mmol/L at baseline was determined to have both the best sensitivity and specificity. A previous study also reported that, during hypertension therapy, predictive factors for developing moderate hyperkalemia (>5.5 mmol/L) under MRA treatment were eGFR <45 mL/min/1.73 m^2^ and baseline serum potassium >4.5 mmol/L [40]: this is consistent with our findings. Such data are easy to use in clinical practice, especially when hyperkalemia is feared: in our study, among patients who experienced at least one episode of hyperkalemia (>5 mmol/L), only two had serum potassium levels at baseline that were lower than this cut-off value, defining a negative predictive value of close to 90%.

The beneficial effects of MRAs have been well established during heart failure, with strong data obtained during randomized controlled trials, especially when cardiac ejection fraction is reduced [41]: both eplerenone and spironolactone have demonstrated improved survival benefits. During chronic kidney disease, the use of MRAs is associated with reducing proteinuria [42]. As proteinuria is one of the most common predictive factors for the progression of kidney disease [43], a beneficial effect of MRAs on kidney survival could be expected but has not been demonstrated previously, due to a lack of randomized controlled trials with kidney survival as the primary endpoint. MRAs could also be useful during CIN [44]: both drugs (MRAs and CsA) act on vascular function. MRAs could limit CsA-induced vascular toxicity. Several animal studies suggest a beneficial effect of MRA use under CsA treatment [9, 10, 13]. It could be related to vascular MR-induced remodeling [45]. To date, no study was published using MRAs and including tacrolimus-treated patients.

The beneficial effects of MRAs could be related to their diuretic effects or their pleiotropic actions (tissue remodeling), as occurs during heart failure [41]. In our study, no effect was observed on systolic, diastolic or mean blood pressure nor on body weight. This is consistent with previous studies: during-end stage renal disease in anuric hemodialyzed patients, MRAs use was effective in reducing mortality without causing a diuretic effect [46], and post-hoc analysis of the EPHESUS trial showed that the beneficial effects of eplerenone were independent of diuretic effects [47].

In our study, MRA dosage was low but was efficient enough at increasing serum potassium. Dose-efficiency has been demonstrated for both spironolactone [48] and eplerenone [49] in reducing morbi-mortality. Moreover, electrolyte disturbances (hyperkalemia) appear to be also dose-dependent [41]. Survival benefits in heart failure have been shown with low posologies: the means were 26 mg/d for spironolactone during the RALES trial [19] and 42 mg/d and 39 mg/d for eplerenone during the EPHESUS [6] and EMPHASIS-HF [5] trials, respectively. Such a low dose has been shown to be efficient during end-stage renal disease in reducing mortality in hemodialyzed patients [46] and morbidity in peritoneal dialysis patients [50]. All these data are consistent with the dosage we chose in the present study: it appeared to be the best compromise between achieving higher efficacy and lower toxicity in our population. Further studies should test the safety and efficiency of higher doses (50 mg/d) that should be facilitated by the use of potassium binders [51].

Taken together, our data show the safe use of eplerenone in CsA-treated transplant recipients, despite renal impairment. This is consistent with a previous study in another population of chronic kidney-disease patients [15]. Of note, our study is the first performed on kidney-transplant recipients.

Further studies are needed to analyze the potential benefits of MRAs in kidney-allograft transplantation: an adequately powered prospective randomized controlled trial should test the efficiency (and safety) of eplerenone in reducing chronic renal-allograft dysfunction, and the potential benefits to survival.

## Supporting Information

S1 Fig CONSORT/TREND statement checklist. To improve the quality of nonrandomized trials, this checklist helped verifying all items.
(PDF)

S2 Fig Trial protocol (English). All the extended methods used in this trial are available here in English and was approved by legal authorities.
(PDF)

S3 Fig Trial protocol (French). All the extended methods used in this trial are available here in French and was approved by legal authorities.
(PDF)
