# Supplementary material for: Safety of Eplerenone for Kidney-Transplant Recipients with Impaired Renal Function and Receiving Cyclosporine A
Source: PLoS One. 2016 Apr 18;11(4):e0153635. doi: 10.1371/journal.pone.0153635 (PMC4835088; doi:10.1371/journal.pone.0153635)
Supplement: S2 Fig — All the extended methods used in this trial are available here in English and was approved by legal authorities. (PDF) [file pone.0153635.s002.pdf]

CHU DE REIMS

AOL
RESEARCH PROTOCOL « MEDICINES »

YEAR 2011

How to use this document:

This document applies to projects on the « Research of Medicines » as defined by the Law for Biomedical Research, and should be using when requesting funding (examples: AOL, National Public Health Research Consortium (PHRC), Interregional PHRC …), in a simplified version which should be completed at the time of submission to the Research Ethics Committee (REC).

This document complies with current regulations; at the time of submission of the file, it is important that you:

- respect the order of the headings and their numbering
- make cross-references to other parts of the document, if necessary,
- write « not applicable », without deleting the heading concerned, if this heading appears unnecessary.

For more information, you can contact research coordination by email at:
coordinationRC@chu-reims.fr

CONTENT

1. GENERAL INFORMATION 4

2. SCIENTIFIC JUSTIFICATION AND GENERAL DESCRIPTION OF THE RESEARCH..8

2.1. NAME AND DESCRIPTION OF THE EXPERIMENTAL DRUGS..8
SUMMARY OF THE RESULTS OF NON-CLINICAL AND CLINICAL TRIALS THAT ARE RELEVANT TO THE BIOMEDICAL RESEARCH..8
2.3. SUMMARY OF THE BENEFITS (IF APPLICABLE) AND THE EXPECTED AND KNOWN RISKS FOR SUBJECTS TAKING PART IN THE STUDY..9
2.4. DESCRIPTION AND JUSTIFICATION FOR THE ROUTE OF ADMINISTRATION, THE DOSE, THE ADMINISTRATION SCHEME AND THE DURATION OF EXPERIMENTAL TREATMENTS 9
2.5. DECLARATION INDICATING THAT THE RESEARCH WILL CONFORM TO THE PROTOCOL, GOOD CLINICAL PRACTICE AND CURRENT LAWS AND REGULATIONS 10
2.6. DESCRIPTION OF THE STUDY POPULATION 10
2.7. REFERENCES TO RELEVANT DATA FROM THE SCIENTIFIC LITERATURE SERVING AS REFERENCES FOR THE STUDY 11

3. STUDY OBJECTIVES 14

3.1. PRIMARY OBJECTIVE 14
3.2. SECONDARY OBJECTIVES 14
3.3. OBJECTIVES OF ANCILLARY STUDES, IF APPLICABLE...14

4. STUDY CONCEPTION 14

4.1. EVALUATION CRITERIA 14
4.2. DESCRIPTION OF THE STUDY METHODOLOGY 15
4.3. DESCRIPTION OF THE MEASURES TAKEN TO REDUCE AND AVOID BIAS.........................17
4.4. EXPERIMENTAL DRUGS..17
4.5. EXPECTED DURATION OF PARTICIPATION OF SUBJECTS AND DESCRIPTION OF THE CHRONOLOGY AND DURATION OF ALL PERIODS OF THE STUDY (INCLUDING FOLLOW-UP) 18
4.6. RULES FOR PERMANENT OR TEMPORARY WITHDRAWAL FROM THE STUDY..........................18
4.7 PROCEDURES FOR ACCOUNTING OF THE EXPERIMENTAL DRUGS..19
4.8 ARRANGEMENTS FOR THE MAINTENANCE OF BLINDING AND PROCEDURE FOR THE LIFTING OF BLINDING, IF RELEVANT 19
4.9 IDENTIFICATION OF ALL DATA TO BE COLLECTED DIRECTLY FROM THE OBSERVATION FILES, WHICH WILL BE CONSIDERED AS A DATA SOURCE 19
4.10 INDEPENDENT SURVEILLANCE COMMITTEE 19

5. SELECTION OF SUBJECTS AND EXCLUSION FROM THE STUDY POPULATION 20

5.1. INCLUSION CRITERIA 20
5.2. EXCLUSION CRITERIA 20
5.3. PROCEDURE FOR THE PREMATURE TERMINATION OF TREATMENT, CORRESPONDING TO STOPPING TREATMENT WITH THE EXPERIMENTAL DRUG, AND PROCEDURE FOR EXCLUSION FROM THE STUDY, CORRESPONDNG TO THE TERMINATION OF TREATMENT AND FOLLOW-UP OF SUBJECTS IN THE CONTEXT OF THIS STUDY 20

6. TREATMENT ADMINISTERED TO SUBJECTS TAKING PART IN THE STUDY...21

6.1. DESCRIPTION OF TREATMENT(S) NECESSARY FOR THE REALISATION OF THE STUDY 21
6.2. ASSOCIATED MEDICINES AND TREATMENTS ALLOWED OR FORBIDDEN BY THE PROTOCOL, INCLUSING RESCUE MEDICATIONS 21

6.3. METHODS OF FOLLOW-UP AND COMPLIANCE WITH TREATMENT 22
6.4. CONDITIONS FOR THE STORAGE OF THE EXPERIMENTAL DRUGS 22

7. EVALUATION OF EFFICACY 22

7.1. DESCRIPTION OF THE PARAMETERS FOR THE EVALUATION OF EFFICACY (EVALUATION CRITERIA) 22
7.2. METHODS AND EXPECTED TIMETABLE FOR THE MEASUREMENT, COLLECTION AND ANALYSIS OF PARAMETERS FOR THE EVALUATION OF EFFICACY 22

8. EVALUATION OF SAFETY 22

8.1. DESCRIPTION OF THE PARAMETERS FOR THE EVALUATION OF SAFETY 22
8.2. METHODS AND EXPECTED TIMETABLE FOR THE MEASUREMENT, COLLECTION AND ANALYSIS OF PARAMETERS FOR THE EVALUATION OF SAFETY 22
8.3. PROCEDURES FOR THE RECORDING AND NOTIFICATION OF ADVERSE EVENTS 23
8.4. METHODS AND DURATION OF FOLLOW-UP FOR SUBJECTS FOLLOWING THE DEVELOPMENT OF AN ADVERSE EVENTS 25

9. STATISTICS 26

9.1. DESCRIPTION OF THE STATISTICAL METHODS PLANNED WITH THE TIMETABLE FOR ANY INTERMEDIATE ANALYSES IF APPLICABLE 26
9.2. NUMBER OF SUBJECTS TO INCLUDE IN THE STUDY WITH STATISTICAL JUSTIFICATION AND THEORETICAL NUMBER OF SUBJECTS EXPECTED AT EACH PLACE OF STUDY, IF APPLICABLE 26
9.3. SIZE OF THE SAMPLE / POPULATION 26
9.4. STATISTICAL METHODS FOR THE ANALYSIS OF THE EVALUATION CRITERIA 27
9.5. TREATMENT OF MISSING DATA 28
9.6. MANAGEMENT OF DATA FOR THE SUBJECTS 28

10. RIGHT OF ACCESS TO THE DATA AND SOURCE DOCUMENTS 28

11. QUALITY CONTROL AND ASSURANCE 28

12. ETHICAL CONSIDERATIONS 29

12.1. LAW FOR BIOMEDICAL RESEARCH AND BIOETHICS 29
12.2. BENEFIT/RISK RATIO 29
12.3. INFORMATION LETTER AND CONSENT FORM FOR THE SUBJECTS 29

13. TREATMENT OF DATA AND STORAGE OF DOCUMENTS AND DATA RELATING TO THE STUDY 35

13.1. RECORDING, COLLECTION AND TREATMENT OF DATA 35
13.2. COLLECTION OF DATA 35
13.3. LOGISTICS AND TREATMENT OF DATA 35
13.4. STORAGE OF DOCUMENTS AND DATA 35

14. FUNDING AND INSURANCE 35

15. RULES RELATING TO PUBLICATION 35

16. LIST OF ANNEXES 36

16.1. ANNEXE 1 : LIST OF INVESTIGATORS 36
16.2. ANNEXE 2 : LIST OF SCIENTIFIC COLLABORATORS, IF APPLICABLE 36

17. SIGNATURE PAGES 37

1. GENERAL INFORMATION

**N° of EUDRACT**	2011, 003759 20
**Promotor and coordinators**	CHU, Reims – 45, rue Cognacq-Jay – 51092 REIMS Cedex represented by its Director, Monsieur Jean-Paul MICHELANGELI
**Title of the study**	STUDY ON THE SAFETY OF EPLERENONE IN RENAL TRANSPLANT PATIENTS TREATED WITH CYCLOSPORINE
**Sort title**	EpleCsAT Safety
**Study code**	AOL2011-06
**Type of study**	☑ Trial of a medicine<br>Safety study
**Investigator - coordinator**	Pr. Philippe RIEU
**Service and Address**	Nephrology Service – Haemodialysis – Transplantation<br>Hôpital Maison Blanche – CHU, Reims<br>Avenue Cognacq Jay<br>51092 Reims CEDEX<br>France
**Investigators and associated centres** *(if applicable)*	Pr. Philippe RIEU<br>Dr Sylvie LAVAUD<br>Dr Olivier TOUPANCE<br>Dr Vincent VUIBLET
**Scientific collaborators**	Jean-Philippe BERTOCCHIO (Intern DES Nephrology)<br>Dr Frédéric JAISSER (Director of Research, INSERM)<br>Pr. Pierre NAZEYROLLAS
**Independent surveillance committee**	☑Yes ❑No
**Theoretical date of the start of the study** September 2011	
**Theoretical date of the end of the study** April 2012	
**Duration of participation of a patient** from 4 to 6 months	
**Number of subjects to include in the study** 31	

Research relevant to the Law for Biomedical Research:

☑Yes ❑ No

If yes, brief description of the benefit/risk ratio

Potential long-term benefit of increasing the survival of a renal transplant and therefore the survival of renal transplant patients; close surveillance study of the adverse effects of the molecule of interest and therefore risk controlled to a maximum.

Summary

Name of the authors and services concerned

Bertocchio JP[1,2], Nazeyrollas P[3], Jaisser F[2], Rieu P[1]

[1] Nephrology Service – Haemodialysis – Transplantation, Hôpital Maison Blanche, CHU de Reims, Avenue du Général Koenig, 51092 Reims CEDEX

[2] INSERM U872, Equipe 1, Centre de Recherche des Cordeliers, 15 rue de l'Ecole de Médecine 75006 Paris CEDEX

[3] Centre for Research, Clinical Investigation and Methodological Assistance, CHU de Reims, Avenue du Général Koenig, 51092 Reims CEDEX

Title of the study

STUDY OF THE SAFETY OF EPLERENONE IN RENAL TRANSPLANT PATIENTS TREATED WITH CYCLOSPORINE

Objectives

To evaluate the safety of eplerenone in association with cyclosporine in renal transplant patients.

Materials and Methods

Principal evaluation criterion: Evaluation of the risk of experiencing an adverse event leading to the termination of treatment. These adverse events are: hyperkalaemia ≥6 mmol/L, hyperkalaemia ≥5.5 mmol/L with 2 spoon-measures/day of KAYEXALATE®, acidosis with alkaline reserve ≤15 mmol/L, arterial hypotension with SAP <100 mmHg, acute renal failure defined as an increase in creatininaemia of >30%.

Secondary criteria: Effect of eplerenone on kalaemia, renal function (GFR measured by the clearance of iohexol, proteinuria, urine and plasma concentrations of NGAL, urinary excretion of TGF-β1, collagen IV and MCP-1) and diuretic effect (fractional excretion of sodium, weight, arterial pressure).

Experimental scheme

Monocentric phase II study conducted in a similar way to the model of Simon: the study will include 3 steps of 8 weeks during which the dose of eplerenone will be increased from 25 to 50 mg/day.

Population / patients

Inclusion criteria: Renal transplant received at least 2 years previously, treated with cyclosporine and with stable renal function between 30 and 50 mL/min/1.73m².

Exclusion criteria: Kalaemia ≥5 mmol/L at inclusion, history of hyperkalaemia ≥6 mmol/L, receiving treatment with a potassium exchange resin, systolic arterial pressure ≤110 mmHg.

Investigational plan

3 steps of 8 weeks during which the dose of eplerenone will be increased from 25 to 50 mg/day. Surveillance will be clinical (the first day of each step, then monthly) and biological (specimens on D0 and D2, and then weekly for each step).

Conduct of study

A steering committee, consisting of the investigators and methodologists from the CHU, Reims, will be directed by the principal investigator (Pr. Rieu) in order ensure the smooth progress of the study.

Timetable

Start of step 1: September 2011 and prospective inclusions with sequential succession to steps 2 and 3

End of study: March 2011 depending on the interval between inclusions.

Plan for the statistical analysis

According to data in the literature, the probabilities of hyperkalaemia occurring during 8 weeks of treatment with eplerenone are <7% with a dose of 25 mg and <10% with a dose of 50 mg in non-transplanted patients with an eGFR >50 mL/min. We posed the alternative hypothesis that the study should be stopped if the risk of poor tolerance of treatment is >15% with the 25 mg dose and 20% with the 50 mg dose. The power to detect an adverse event requiring the termination of treatment was fixed at 90%. The number of subjects necessary to include is 31 patients.

TYPE OF RESEARCH

Tick the appropriate boxes

IF YOUR RESEARCH IS ORGANISED AND PRACTISED ON HUMANS WITH THE AIM OF DEVELOPING SPECIFIC BIOLOGICAL OR MEDICAL KNOWLEDGE

IF YOUR RESEARCH IS DIRECTED AT:

- Innovating techniques or strategies;

YES ☐ NO ☑

- Techniques or strategies considered to be obsolete;

YES ☐ NO ☑

- On the evaluation of an innovating combination of procedures or products, even if each of these is currently used independently

YES ☑ NO ☐

- On a comparison of medical strategies, when one of these strategies may, according to our current state of knowledge, be considered superior to the other in terms of safety or efficacy

YES ☐ NO ☑

IF THE OBJECTIVE OF YOUR RESEARCH IS:

- To evaluate acts, combinations of acts or medical strategies for prevention, diagnosis or treatment, which are practised currently and are the object of professional consensus in respect to their indications.

YES ☐ NO ☑

NB: Attention: if the case above is ticked « yes », your protocol comes under Current Care and you must change file.

2. SCIENTIFIC JUSTIFICATION AND GENERAL DESCRIPTION OF THE RESEARCH

2.1. Name and description of the experimental drugs

Eplerenone (INSPRA®) is a selective antagonist of mineralocorticoid receptors (MR). It already has marketing authorisation (MA) in France for its beneficial effect on survival in heart failure. It is administered orally, generally at a dose of 50 mg/day. The half-life of eplerenone is between 4 and 6 hours (Struthers *et al.*, 2008).
Cyclosporine A (NEORAL®) (CsA) is a powerful immunosuppressant used since the 1980s in organ transplantation. It is an antirejection treatment, of the anti-calcineurin family, which induces a decrease in interleukin 2 synthesis in T-lymphocytes leading to a phenomenon of tolerance on the part of the host immune system vis-à-vis the transplant. CsA has MA in the context of renal transplantation. It is an oral treatment, generally used at a dose of 2 to 6 mg/kg/day divided into 2 doses (at 12 h intervals), whose dose is adapted to the concentration of the parent compound and area under the curve.

Summary of the results of non-clinical and clinical trials that are relevant to the biomedical research

Experimental data

<u>MR antagonists reduce the renal toxicity of cyclosporine:</u>
In a model of acute nephrotoxicity in rats, Perez-Rojas *et al.* demonstrated that spironolactone (a MR antagonist) could reverse the renal damage caused by CsA mediated by vaso-active factors (Perez-Rojas *et al.*, 2005). Furthermore, our group demonstrated that canrenoate (a metabolite of spironolactone) not only prevented mortality induced by CsA in mice but also prevented the change in renal function and lesions of tubular vacuolization (Bertocchio *et al.*, 2010a, Bertocchio *et al.*, 2010b).
In terms of chronic damage in rats, spironolactone not only prevent mortality (Perez-Rojas *et al.*, 2005) but also weight loss (Perez-Rojas *et al.*, 2005, Macunluoglu *et al.*, 2008). Moreover, renal function was partially preserved (Perez-Rojas *et al.*, 2005, Macunluoglu *et al.*, 2008, Feria *et al.*, 2003). Histologically, afferent arteriolopathy was prevented (Feria *et al.*, 2003) as well as direct fibrosis (Feria *et al.*, 2003) or via mediators of fibrosis (Macunluoglu *et al.*, 2008). At the molecular level, in these models, spironolactone modulates the expression of mRNA (messenger RMA) of vaso-active factors (Perez-Rojas *et al.*, 2005) and fibrosis (Feria *et al.*, 2003). These effects seem to be shared by molecules of the same therapeutic class (MA antagonists) because similar efficacy was found in rats with another antagonist: the administration of eplerenone simultaneously with CsA significantly prevented an increase in arterial pressure, weight loss, an alteration in renal function, a decrease in renal blood flow, an alteration of lithium clearance and histological renal lesions (tubular vacuolization and vascular deposits) (Nielsen *et al.*, 2008, Nielsen *et al.*, 2010).
Furthermore, a publication of a study in rats seemed to suggest that the lesions of chronic nephrotoxicity could be partially reversible with spironolactone: renal function, tubulo-interstitial fibrosis, afferent arteriolopathy, glomerular ischaemia and apoptosis were reversible after the development of these lesions (Waanders *et al.*, 2009).
In the first model of allogeneic renal transplantation in rats, Waanders *et al.* (2009) demonstrated that MR antagonism decreased inflammation, fibrosis and allograft vasculopathy.

Clinical trials

No study analysing the interest of MR antagonists on the prevention of CsA nephrotoxicity in humans has been published to date.

Only one study, in a transplanted patient with proteinuria despite double-blockade of the Renin-Angiotensin-Aldosterone System (RAAS) (by an angiotensin-converting enzyme inhibitor (ACE inhibitor) and Angiotensin II Receptor Antagonist (ARA2)) reported the efficacy of the addition of spironolactone on proteinuria (Gonzalez Monte *et al.*, 2010).

2.3. Summary of the benefits (if applicable) and the expected and known risks for subjects taking part in the study

The expected benefits for patients participating in this study, taking into account its short duration, are small.
The expected benefits for society are essentially the possibility of carrying out a further study on the efficacy of eplerenone for the prevention of CsA nephrotoxicity. Currently, there are no drugs that can limit the renal toxicity of CsA. The aim of this future study will be to investigate the effect of eplerenone on the loss of renal function of renal transplant patients treated with CsA: the effect could be a longer life-expectancy of renal transplants and thus a lower cost (compared to a return to extra-renal dialysis or even a new transplant). These beneficial effects could be shared by patients participating in a prospective study comparing eplerenone with placebo.
The adverse events linked to the use of MR antagonists can be divided into ionic effects (hyperkalaemia and hydrosodium depletion via their diuretic effect) and anti-androgenic affects (gynecomastia, problems with the menstrual cycle, impotence, etc…) (Bertocchio and Jaisser, 2010, Bertocchio *et al.*, 2011). The use of MR antagonists is usually not advised in patients with renal failure due to the increased risk of hyperkalaemia, particularly if another RAAS antagonist is added.
In France, there are two MR antagonists available on the market: spironolactone and eplerenone. The half-life of spironolactone is between 14 and 16 hours while that of eplerenone is between 4 and 6 hours. These two molecules are mainly metabolised by the liver after primary hepatic passage, but the metabolites of spironolactone also have a pharmacological effect by antagonising MR, glucocorticoid receptors and androgenic receptors (Struthers *et al.*, 2008, Epstein, 2003). Spironolactone has a strong affinity for MR, but eplerenone is more selective for MR. This selectivity is linked to the structure of the molecule (Sica, 2005). *In vitro*, nearly 400-times more eplerenone is required than spironolactone to antagonise other steroid receptors such as glucocorticoid receptors, androgen receptors and progesterone receptors, which are implicated in the development of adverse events in humans (Garthwaite and McMahon, 2004). The adverse effects of these two molecules have been compared in a randomised double-blind study (Parthasarathy *et al.*, 2011): the anti-androgenic effects, which are regularly a cause of termination of treatment, are clearly more frequent with spironolactone than with eplerenone.
Finally, a recent study highlighted an effect of spironolactone on glucidic metabolism that is not present with eplerenone: through its antagonistic action on the glucocorticoid receptor, spironolactone slightly increases cortisolaemia, thus exposing patients to glycaemic imbalance. In a prospective study of more than 100 patients, eplerenone and spironolactone were compared for criteria relating to glucidic metabolism; in contrast to eplerenone, spironolactone significantly increased cortisolaemia, the plasma concentration of adiponectin and glycosylated haemoglobin (HbA1c) (Yamaji *et al.*, 2010). Renal transplant patients, particularly those receiving CsA and/or corticoids, have an increased risk of diabetes (or NODAT, New-Onset Diabetes Mellitus After Transplantation). Although it has not been demonstrated that spironolactone induces diabetes, it appears wise to use a molecule that has less disruptive effect on glucidic metabolism in these patients.

2.4. Description and justification for the route of administration, the dose, the administration scheme and the duration of experimental treatments

Eplerenone was granted MA for oral administration after the EPHESUS study (Pitt *et al.*, 2003) which demonstrated its efficacy in heart failure. It is administered daily as one dose.

In this tolerance study, we wish to investigate increasing doses of the two commercially available preparations (25 and 50 mg) in order to determine the therapeutic window between the minimal effective dose and the maximum tolerated dose (limiting this to a maximum of 50 mg/day) in renal transplant patients receiving CsA.
In a Spanish study in 11 patients (Gonzalez Monte *et al*., 2010), 25 mg spironolactone achieved the primary objective of efficacy (proteinuria) in 3 months. The effect on kalaemia was maximal from the first month of treatment. We therefore think that we will see an increase in kalaemia in the first weeks of treatment (which will be maximal at the 4th week) and other adverse events in the following weeks: for this reason, we will carry out steps of 8 weeks (2 months).

2.5. Declaration indicating that the research will conform to the protocol, good clinical practice and current laws and regulations

If the study is funded, the investigators agree to comply with the protocol for clinical research, good clinical practice and current laws and regulations.

2.6. Description of the study population

In this tolerance study, we wish to determine the therapeutic window between the minimum effective dose and the maximum tolerated dose (limiting this to 50 mg/day maximum) in renal transplant patients receiving CsA with altered renal function (with an eGFR between 30 and 50 mL/min/1.73m²).
The use of eplerenone is currently contraindicated in patients with renal failure defined by a creatinine clearance of <50 mL/min/1.73m² because there have been no specific studies in this population. However, a large multicentre study in cardiac failure (Zannad *et al*., 2011) recently investigated the effect of eplerenone from stage II of the NYHA classification: nearly one-third of patients presented with chronic renal failure with an eGFR <60 mL/min/1.73m².
Similarly, the association of eplerenone with CsA is usually contraindicated because there has been no specific study in this population. By carrying out a study in patients receiving CsA with altered renal function, we will have the best conditions to reveal the major adverse event anticipated: hyperkalaemia.
We will select patients who have been transplanted for more than 2 years because this is when the doses of immunosuppressants and renal function are stable. Furthermore, from the end of the 2nd year of transplantation, the risk of acute rejection is low. We will exclude, for the same reasons, patients who have had an episode of acute rejection in the year prior to the date of inclusion. Furthermore, at 2 years after transplantation, nearly 50% of patients have histological signs of CsA toxicity (Nankivell *et al*., 2003).
At an eGFR of <30 mL/min/1.73m², the lesions caused by CsA toxicity do not appear to be reversible. If the adverse effects of eplerenone are absent or acceptable, this molecule could be proposed to patients treated with CsA with a renal function >30 mL/min/1.73m².

Patients able to participate in the study should fulfil the following criteria (inclusion criteria):
- age ≥18 years on the date of inclusion
- possess full legal competence
- give their written consent
- be affiliated to a Social Security Regime
- have had a functional renal transplant for ≥2 years at the date of inclusion
- receiving treatment with CsA
- have a renal function estimated by the MDRD equation (Levey *et al*., 1999) between 30 and 50 mL/min/1.73m²

Patients able to participate in the study should not fulfil any of the following criteria (exclusion criteria):

- kalaemia ≥5 mmol/L at inclusion
- one or more previous episodes of severe hyperkalaemia (≥6 mmol/L), whatever the cause
- be receiving treatment with a potassium exchange resin such as KAYEXALATE®
- an episode of acute rejection during the 12 months prior to inclusion
- be pregnant or not using effective contraception during the study
- uncontrolled arterial hypertension
- orthostatic hypotension
- systolic arterial pressure ≤110 mmHg
- cardiac failure in the 3 months prior to inclusion or cardiac failure (NYHA stages III or IV)
- severe liver failure (Child-Pugh class C)
- allergy to one of the components of eplerenone – INSPRA®
- be receiving treatment with spironolactone – ALDACTONE® or eplerenone – INSPRA®
- have a contraindication for which treatment cannot be suspended during the study: diuretic for hyperkalaemia, potassium salts, CYP3A4 enzyme inhibitor (such as itraconazole, ketoconazole, ritonavir, nelfinavir, clarithromycin, telithromycin, nefazodone)
- be receiving treatment with trimethoprim or digoxin
- be receiving treatment with alpha 1 blockers
- be receiving treatment with warfarin
- a malabsorption syndrome, an anomaly of galactose metabolism, galactase deficiency
- be receiving treatment with a non-steroidal anti-inflammatory drug, a lithium-based treatment, or any other nephrotoxic treatment
- be receiving treatment with double-blockade of the RAAS by the association ACE inhibitor and ARA2
- be a protected patient (under supervision, guardianship, placed under the protection of the law..)

2.7. References to relevant data from the scientific literature serving as references for the study

BERTOCCHIO, J. P., DUONG VAN HUYEN, J. P., EL MOGHRABI, S., RIEU, P. & JAISSER, F. (2010a) Beneficial effect of mineralocorticoid receptor antagonism in acute cyclosporine A nephrotoxicity. *American Society of Nephrology - Renal Week.* Denver.

BERTOCCHIO, J. P., DUONG VAN HUYEN, J. P., EL MOGHRABI, S., RIEU, P. & JAISSER, F. (2010b) Implication de l'activation du récepteur minéralocorticoïde dans la néphrotoxicité aiguë de la cyclosporine A. *12ème Réunion Commune de la Société de Néphrologie et de la Société Francophone de Dialyse.* Bruxelles.

BERTOCCHIO, J. P. & JAISSER, F. (2010) [Aldosterone and kidney diseases: An emergent paradigm with important clinical implications.]. *Nephrol Ther.*

BERTOCCHIO, J. P., WARNOCK, D. G. & JAISSER, F. (2011) Mineralocorticoid receptor activation and blockade: an emerging paradigm in chronic kidney disease. *Kidney Int,* 79**,** 1051-60.

EPSTEIN, M. (2003) Aldosterone receptor blockade and the role of eplerenone: evolving perspectives. *Nephrol Dial Transplant,* 18**,** 1984-92.

FERIA, I., PICHARDO, I., JUAREZ, P., RAMIREZ, V., GONZALEZ, M. A., URIBE, N., GARCIA-TORRES, R., LOPEZ-CASILLAS, F., GAMBA, G. & BOBADILLA, N. A. (2003) Therapeutic benefit of spironolactone in experimental chronic cyclosporine A nephrotoxicity. *Kidney Int,* 63**,** 43-52.

GARTHWAITE, S. M. & MCMAHON, E. G. (2004) The evolution of aldosterone antagonists. *Mol Cell Endocrinol,* 217**,** 27-31.

GONZALEZ MONTE, E., ANDRES, A., POLANCO, N., TORIBIO, M. J., SANTANA, R., GUTIERREZ MARTINEZ, E., GONZALEZ, J., RAMIREZ, E., HERNANDEZ, A., MORALES, E., PRAGA, M. & MORALES, J. M. (2010) Addition of spironolactone to dual blockade of renin angiotensin system dramatically reduces severe proteinuria in renal transplant patients: an uncontrolled pilot study at 6 months. *Transplant Proc,* 42**,** 2899-901.

LEVEY, A. S., BOSCH, J. P., LEWIS, J. B., GREENE, T., ROGERS, N. & ROTH, D. (1999) A more accurate method to estimate glomerular filtration rate from serum creatinine: a new prediction equation. Modification of Diet in Renal Disease Study Group. *Ann Intern Med,* 130**,** 461-70.

MACUNLUOGLU, B., ARIKAN, H., ATAKAN, A., TUGLULAR, S., ULFER, G., CAKALAGAOGLU, F., OZENER, C. & AKOGLU, E. (2008) Effects of spironolactone in an experimental model of chronic cyclosporine nephrotoxicity. *Transplant Proc,* 40**,** 273-8.

NANKIVELL, B. J., BORROWS, R. J., FUNG, C. L., O'CONNELL, P. J., ALLEN, R. D. & CHAPMAN, J. R. (2003) The natural history of chronic allograft nephropathy. *N Engl J Med,* 349**,** 2326-33.

NIELSEN, F. T., JENSEN, B. L., HANSEN, P. B. & BIE, P. (2010) A Mineralocorticoid Receptor Antagonist Prevents Chronic Cyclosporinee A Nephropathy in the Rat. *American Society of Nephrology - Renal Week.* Denver.

NIELSEN, F. T., JENSEN, B. L., MARCUSSEN, N., SKOTT, O. & BIE, P. (2008) Inhibition of mineralocorticoid receptors with eplerenone alleviates short-term cyclosporine A nephrotoxicity in conscious rats. *Nephrol Dial Transplant,* 23**,** 2777-83.

PARTHASARATHY, H. K., MENARD, J., WHITE, W. B., YOUNG, W. F., JR., WILLIAMS, G. H., WILLIAMS, B., RUILOPE, L. M., MCINNES, G. T., CONNELL, J. M. & MACDONALD, T. M. (2011) A double-blind, randomized study comparing the antihypertensive effect of eplerenone and spironolactone in patients with hypertension and evidence of primary aldosteronism. *J Hypertens,* 29**,** 980-90.

PEREZ-ROJAS, J. M., DERIVE, S., BLANCO, J. A., CRUZ, C., MARTINEZ DE LA MAZA, L., GAMBA, G. & BOBADILLA, N. A. (2005) Renocortical mRNA expression of vasoactive factors during spironolactone protective effect in chronic cyclosporine nephrotoxicity. *Am J Physiol Renal Physiol,* 289**,** F1020-30.

PITT, B., REMME, W., ZANNAD, F., NEATON, J., MARTINEZ, F., RONIKER, B., BITTMAN, R., HURLEY, S., KLEIMAN, J. & GATLIN, M. (2003) Eplerenone, a selective aldosterone blocker, in patients with left ventricular dysfunction after myocardial infarction. *N Engl J Med,* 348**,** 1309-21.

PITT, B., ZANNAD, F., REMME, W. J., CODY, R., CASTAIGNE, A., PEREZ, A., PALENSKY, J. & WITTES, J. (1999) The effect of spironolactone on morbidity and mortality in patients with severe heart failure. Randomized Aldactone Evaluation Study Investigators. *N Engl J Med,* 341**,** 709-17.

SICA, D. A. (2005) Pharmacokinetics and pharmacodynamics of mineralocorticoid blocking agents and their effects on potassium homeostasis. *Heart Fail Rev,* 10**,** 23-9.

SIMON, R. (1989) Optimal two-stage designs for phase II clinical trials. *Control Clin Trials,* 10**,** 1-10.

STRUTHERS, A., KRUM, H. & WILLIAMS, G. H. (2008) A comparison of the aldosterone-blocking agents eplerenone and spironolactone. *Clin Cardiol,* 31**,** 153-8.

WAANDERS, F., RIENSTRA, H., BOER, M. W., ZANDVOORT, A., ROZING, J., NAVIS, G., VAN GOOR, H. & HILLEBRANDS, J. L. (2009) Spironolactone ameliorates transplant vasculopathy in renal chronic transplant dysfunction in rats. *Am J Physiol Renal Physiol,* 296**,** F1072-9.

YAMAJI, M., TSUTAMOTO, T., KAWAHARA, C., NISHIYAMA, K., YAMAMOTO, T., FUJII, M. & HORIE, M. (2010) Effect of eplerenone versus spironolactone on cortisol and hemoglobin A(c) levels in patients with chronic heart failure. *Am Heart J,* 160**,** 915-21.

ZANNAD, F., MCMURRAY, J. J., KRUM, H., VAN VELDHUISEN, D. J., SWEDBERG, K., SHI, H., VINCENT, J., POCOCK, S. J. & PITT, B. (2011) Eplerenone in patients with systolic heart failure and mild symptoms. *N Engl J Med,* 364**,** 11-21.

3. STUDY OBJECTIVES

3.1. Primary objective

This is a tolerance study aimed at determining whether tolerance to eplerenone, in a renal transplant patient treated with CsA and with an eGFR between 30 and 50 mL/min/1.73m², will allow the use of this molecule at a dose of 25 or 50 mg/day.

3.2. Secondary objectives

The secondary objectives are to carry out a preliminary evaluation on the effect of eplerenone in this population on markers of renal function: essentially eGFR and proteinuria.
However, other markers of hydrosodium depletion (weight, fractional sodium excretion) will be studied in order to distinguish the part played by sodium depletion (diuretic effect of eplerenone) from the part played by the specific effects of eplerenone (anti-inflammatory, anti-fibrosis, anti-proteinuric effects, etc…).

3.3. Objective of ancillary studies, if applicable

Subsequently, an ancillary study will evaluate the impact of eplerenone on biomarkers of inflammation (MCP-1), fibrosis (TGF-β1 and collagen IV, for example) or even renal damage (NGAL), which can be measured in the blood and urine of patients taking part in the study. The request made in the context of this local bid ('Appel d'Offre Local' 2011 (AOL 2011)) will not fund this ancillary study and a request for funds will be made specifically for this additional work.

4. STUDY CONCEPTION

4.1. Evaluation criteria

Primary criterion: *appearance of adverse events requiring the termination of treatment*
The adverse events requiring the termination of treatment that will be considered are:
- hyperkalaemia ≥6 mmol/L and/or hyperkalaemia ≥5.5 mmol/L under 2 spoon-measures/day of KAYEXALATE®
- acidosis revealed by an alkaline reserve ≤15 mmol/L
- systemic arterial hypotension revealed by a systolic arterial pressure <100 mmHg
- orthostatic hypotension defined by a decrease in systolic arterial pressure of >20 mmHg in the 3 minutes following standing
- acute renal failure defined by an increase in creatininaemia of >30% compared to its value at inclusion
- all other unexpected adverse events requiring the termination of eplerenone treatment

Secondary criteria:
- use of a potassium exchange resin
- systolic arterial pressure, diastolic, mean
- weight
- renal function (creatininaemia and creatinine clearance calculated using the MDRD formula)
- vascular resistance of the renal transplant
- natraemia, fractional sodium excretion ($FeNa^+$)
- natriuresis, creatininuria, kaluresis
- proteinuria, albuminuria

Criteria for the ancillary study:
- urine and plasma concentrations of NGAL
- urine excretion of TGF-β1, collagen IV and MCP-1

4.2. Description of the study methodology

Study scheme **(Figure 1)**
The tolerance study will be carried out by adapting the 2-step scheme of Simon (Simon, 1989) initially, and then by carrying out an increase in doses if tolerance is satisfactory.
From the data in the literature, it appears that the frequency of intolerance to treatment is <7% for a dose of 25 mg/day and <10% for a dose of 50 mg/day, which seems acceptable in this population and leads us to envisage a subsequent therapeutic trial. Conversely, its use does not appear possible if the respective frequencies are >15% and 20%, respectively.
In these conditions, for this tolerance study, we proposed a scheme which includes three successive stages combining the plan of Simon in two stages at a dose of 25 mg/day, and then an increase in dose to 50 mg/day with evaluation at a single time **(Figure 1)**.
Under these conditions, the risk of not revealing unacceptable tolerance of eplerenone in this population is <10% and the risk of deciding, if tolerance permits, to not use it is 27%. There is a 60% chance of interrupting the study before the increase in dose if the hypothesis of unfavourable treatment is correct.
The advantages of the study scheme are that it will demonstrate unacceptable toxicity as early as possible and will detect a possible threshold effect between the doses of 25 mg/day and 50 mg/day which will allow the use of the lowest dose possible.

Step 1: 14 patients will be included in the first step, at an eplerenone dose of 25 mg/day. This stage will last 8 weeks. If ≥3 adverse events requiring the termination of treatment occur during these 8 weeks, the study will be stopped and the treatment will be considered to be too poorly tolerated to be used in this population. If <3 adverse events occur, we will proceed to step 2.
Step 2: This will consist of the addition of an additional 17 patients. All of the patients (14+17) will be treated with eplerenone at a dose of 25 mg/day for 8 weeks. If >4 adverse events requiring the termination of treatment occur during the 8 weeks, the study will be stopped and the treatment will be considered to be too poorly tolerated to be used in this population. If ≤4 adverse events occur, we will proceed to step 3.
Step 3: This will consist of an increase in the dose of eplerenone to 50 mg/day in all patients who participated in step 2. The duration of this step will be 8 weeks. If ≥5 adverse events requiring the termination of treatment occur, the study will be stopped and the treatment will be considered to be too poorly tolerated to be used in this population. If ≤4 adverse events occur, the tolerance of eplerenone treatment in this population will be considered to be acceptable.

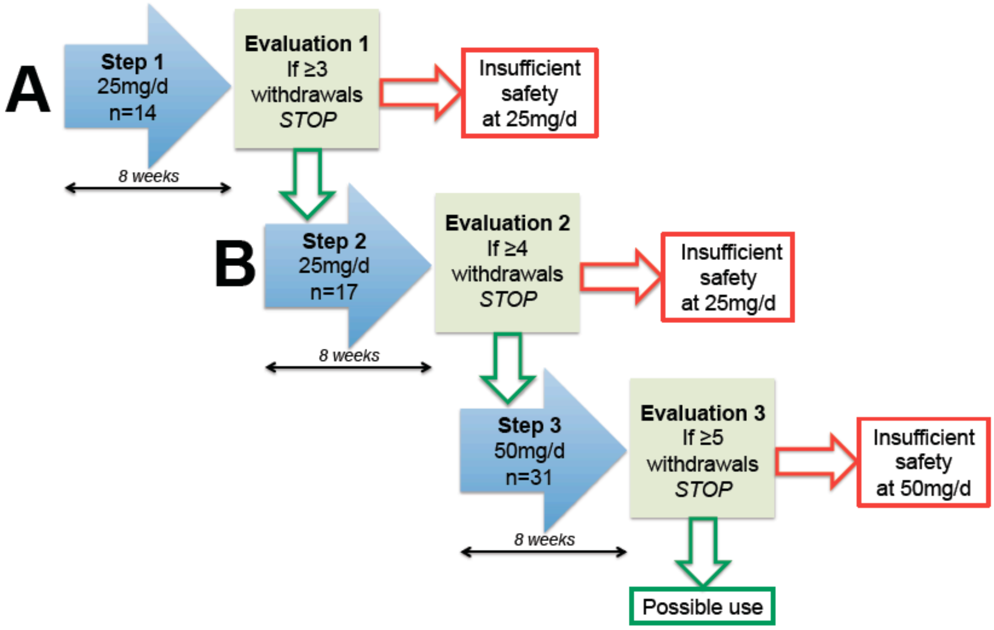

Figure 1. Study scheme showing the different possibilities

***Clinical and biological surveillance* (Tables 1 and 2)**
Clinical surveillance for group A:

- Step 1: D0, D14, D28 and D56.
- Step 2: D0 (D56 of step 1), D28 (84th day of participation) and D56 (112th day of participation)
- Step 3 (change of dose): D0 (D56 of step 2), D14 (126th day of participation), D28 (140th day of participation) and D56 (168th day of participation).

Clinical surveillance for group B:

- Step 2: D0, D14, D28 and D56.
- Step 3 (change of dose): D0 (D56 of step 2), D14 (70th day of participation), D28 (84th day of participation) and D56 (112th day of participation).

Biological surveillance: blood samples on D0, D2, then weekly until the end of this step for kalaemia, blood samples on D0, D14, D28 and then every month for creatininaemia and urine samples on D0 and then every month for creatininuria, blood inogram and proteinuria.
The specimens for the measurement of kalaemia should be taken without a tourniquet.
Monitoring of kalaemia 48 hours after the initiation of treatment (D2) then every 7 days until the end of the study. Consultation with an investigator, in addition to the planned schedule, if necessary (appearance of an adverse event).
The specimens at inclusion, during consultations and at the end of the study will be taken at the Nephrology Service Day Hospital (NSDH) of the CHU, Reims. The specimens for surveillance of kalaemia will be taken at the usual Medical Analysis Laboratory (MAL) used by patient. An agreement will link the investigational centre and the MAL. The result for kalaemia will be faxed to the CRICAM (Fax number: 03 28 78 88 39), and the investigational physician will be informed of the result on the same day).
Two groups will be distinguished:

- Group A will comprise patients who start the study on the first day of the study; there will be 14 at inclusion
- Group B will consist of patients who start the study directly at step 2; there will be 17 who will be included specifically
-

Table 1. Timetable for clinical and biological follow-up of group A

	Etape 1										Etape 2										Etape 3							
	J0	J2	J7	J14	J21	J28	J35	J42	J49	J56 = J0	J2	J7	J14	J21	J28	J35	J42	J49	J56 = J0	J2	J7	J14	J21	J28	J35	J42	J49	J56
Recueil du consentement	x																											
Clinique																												
Pression Artérielle	x			x		x				x					x				x			x		x				x
Recherche d'effets indésirables	x			x		x				x					x				x			x		x				x
Poids	x			x		x				x					x				x			x		x				x
Electrocardiogramme	x					x				x					x				x					x				x
Biologique																												
Fonction rénale estimée (eDFG par MDRD)	x			x		x				x					x				x			x		x				x
Kaliémie	x	x	x	x	x	x	x	x	x	x	x	x	x	x	x	x	x	x	x	x	x	x	x	x	x	x	x	x
Fraction excrétée de sodium ($FeNa^+$)	x					x				x					x				x					x				x
Ionogramme urinaire (NaU, KU), créatininurie, protéinurie	x					x				x					x				x					x				x
Albuminurie	x									x									x									x
Sérothèque	x									x									x									x
Urinothèque	x									x									x									x
Fonctionnel																												
Echo-doppler de greffon	x																											x

Table 2. Timetable for clinical and biological follow-up of group B

	Etape 2										Etape 3								
	J0	J2	J7	J14	J21	J28	J35	J42	J49	J56 = J0	J2	J7	J14	J21	J28	J35	J42	J49	J56
Recueil du consentement	x																		
Clinique																			
Pression Artérielle	x			x		x				x			x		x				x
Recherche d'effets indésirables	x			x		x				x			x		x				x
Poids	x			x		x				x			x		x				x
Electrocardiogramme	x					x				x					x				x
Biologique																			
Fonction rénale estimée (eDFG par MDRD)	x			x		x				x			x		x				x
Kaliémie	x	x	x	x	x	x	x	x	x	x	x	x	x	x	x	x	x	x	x
Fraction excrétée de sodium ($FeNa^+$)	x					x				x					x				x
Ionogramme urinaire (NaU, KU), créatininurie, protéinurie	x					x				x					x				x
Albuminurie	x									x									x
Sérothèque	x									x									x
Urinothèque	x									x									x
Fonctionnel																			
Echo-doppler de greffon	x																		x

4.3. Description of the measures taken to reduce and avoid bias

Random sampling

Not applicable

Methods of blinding

Not applicable

4.4. Experimental drugs

4.4.1 Description of the dose and methods of administration of the experimental drug(s)

Eplerenone 25 mg/day or 50 mg/day as a single dose by mouth to be taken every day of the study: 25 mg/day during steps 1 and 2, then 50 mg/day during step 3.
This treatment will be provided to the patients by the promoting centre. The pharmacy associated with this study is the pharmacy of the CHU, Reims (Hôpital Robert Debré), and the pharmacist responsible for this study is Mr Philippe Benoit.

4.4.2 Description of the unit form, packaging and labelling of the experimental drug(s).

Eplerenone
Medicine commercially available in boxes of 28 tablets, available individually from the hospital pharmacy of the CHU, Reims
Eplerenone is available as a yellow film-coated tablet with the inscription « Pfizer » on one surface and « NSR » and « 25 » (or « 50 » depending on the dose) on the other.

Cyclosporine
Medicine commercially available in boxes of 50 to 60 soft capsules, available individually from the hospital pharmacy of the CHU, Reims. CsA is available as a soft capsule in doses of 10, 25, 50 or 100 mg.

4.5. Anticipated duration of participation of subjects and description of the chronology and duration of all periods of the study (including follow-up)

4.5.1 Expected duration of participation of subjects

The maximum participation of patients taking part in the study in group A will be 3 steps of 8 weeks, thus 24 weeks in total.
The maximum participation of patients taking part in the study in group B will be the 2 final steps of 8 weeks (2 and 3), thus 16 weeks in total.

4.5.2 Description of the chronology and duration of all periods of the study, including follow-up

See section 4.2.

4.6. Rules for permanent or temporary withdrawal from the study

See Figure 1 and section 4.2
Withdrawal from the study is permanent: a patient who has left the study cannot re-enter the study.

4.6.1 For participation of a patient in the study

The participation of a patient finishes either at the end of the study (end of step 3) or after the development of an adverse event requiring the premature termination of eplerenone in this patient.

4.6.2 For all or part of the study

The end of the study will either be at the end of step 3 or after the development of a large number of adverse events requiring the premature termination of eplerenone in the patients as defined in section 4.2.

4.7 Procedures for accounting of the experimental drug(s)

Counting of the packaging by the pharmacy at the end of each step.

4.8 Arrangements for the maintenance of blinding and procedure for the lifting of blinding, if relevant

Not applicable

4.9 Identification of all data to be collected directly from the observation files, which will be considered as source data

The following data will be collected from the observation files:

Demographic data
Date of birth
Sex
Weight
Height
Clinical data
Systolic and diastolic arterial pressure, lying down and standing
Previous history: diabetes, arterial hypertension, obesity, cardiac failure, respiratory failure, myocardial infarction, other
Electrocardiogram: problems with rhythm, problems with conduction, problems with repolarisation
Particularities of the clinical examination: presence of oedema of the lower limbs
Other treatments taken during the study
Appearance of adverse events (anti-androgenic effects in particular)
Biological data
Kalaemia
Creatininaemia, eGFR (according to the MDRD formula)
Natraemia, natriuresis, fractionated sodium excretion
Creatininuria
Kaluresis
Proteinuria, albuminuria
Serum profile, urine profile

Functional data
Vascular resistance of the transplant

4.10 Independent Surveillance Committee

In accordance with article L1123-7 of the Public Health Code, transposing European Directive 2001/20/CE to French law, an Independent Surveillance Committee (ISC) will be set up by the study promotor. The constitution and function of the ISC will conform to the Guidelines on Data Monitoring Committees of the European Medicines Agency (JO n°377 of 30th November 2006). This committee will guarantee the scientific integrity of the study.
The promotor will name 3 independent experts who will meet:

- before the start of the study, in order to approve its method of functioning, the study protocol, the methods for declaring any suspected adverse events and the methods for stopping treatment defined in the protocol
- during the study at least once in order to evaluate the tolerance and efficacy of the study drug as well as to control the general quality and progress of the study
- at the end of the study in order to approve the general quality of the running of the study

Each meeting will consist of a detailed review in which the ISC will give its recommendations: carry on without modification, carry on with modification(s), or even stop the study. These recommendations will be sent to the promotor and to the investigators.
If necessary, the ISC could have access to all of the study data. In this study, all of the files will be monitored by the ISC. It will also be informed of all new data that question the pertinence of the study.

5. SELECTION OF SUBJECTS AND EXCLUSION FROM THE STUDY POPULATION

5.1. Inclusion criteria

Patients able to participate in the study should fulfil the following criteria:
- age ≥18 years at inclusion
- possess full legal capacity
- give their written consent
- be affiliated to a Social Security Regime
- have had a function renal transplant for at least 2 years at inclusion
- be treated with cyclosporine
- presenter a renal function estimated with the MDRD formula (Levey et al., 1999) between 30 and 50mL/min/1.73m²

5.2. Exclusion criteria

Patients able to participate in the study should not fulfil any of the following conditions:
- kalaemia ≥5 mmol/L at inclusion
- one of more previous episodes of severe hyperkalaemia (≥6 mmol/L), whatever the cause
- be receiving treatment with a potassium exchange resin such as KAYEXALATE®
- an episode of acute rejection during the year prior to inclusion
- be pregnant or not using effective contraception during the study
- uncontrolled arterial hypertension
- orthostatic hypotension
- systolic arterial pressure ≤110 mmHg
- cardiac failure in the 3 months prior to inclusion or cardiac failure (NYHA classes III or IV)
- severe liver failure (Child-Pugh class C)
- an allergy to one of the components of eplerenone – INSPRA®
- be receiving treatment with spironolactone – ALDACTONE® or eplerenone – INSPRA®
- have a contraindication for which treatment cannot be stopped during the study: diuretic for hyperkalaemia, potassium salt, CYP3A4 enzyme inhibitor (such as itraconazole, ketoconazole, ritonavir, nelfinavir, clarithromycin, telithromycin, nefazodone)
- be receiving treatment with trimethoprim or digoxin
- be receiving treatment with alpha 1 blockers
- be receiving treatment with warfarin
- a malabsorption syndrome, an anomaly of galactose metabolism, galactase deficiency
- be receiving treatment with a non-steroidal anti-inflammatory drug, a lithium-based treatment, or other nephrotoxic treatment
- be receiving treatment with double-blockade of the RAAS by the association ACE inhibitor and ARA2
- be a protected patient (under supervision, guardianship, under the protection of the law..)

5.3. Procedure for the premature termination of treatment, corresponding to stopping treatment with the experimental drug, and procedure for exclusion

from the study, corresponding to the termination of treatment and follow-up of the subject in the context of the study

The patients taking part in this study cannot participate simultaneously in another therapeutic trial. This ban lasts until 1 month after the end of participation in this study.

5.3.1 Criteria and modalities for the premature termination of treatment or exclusion of a subject from the study

A patient participating in the study leaves the study when they:
1) present with at least one of the adverse event criteria requiring the cessation of eplerenone treatment:
- hyperkalaemia ≥6 mmol/L and/or hyperkalaemia ≥5.5 mmol/L under 2 spoon-measures/day of KAYEXALATE®
- acidosis revealed by an alkaline reserve of ≤15 mmol/L
- systemic arterial hypotension revealed by a systolic arterial pressure <100mHg
- orthostatic hypotension defined by a decrease in systolic arterial pressure of >20 mmHg in the 3 minutes following standing
- acute renal failure defined by an increase in creatininaemia of >30% in comparison with its value at inclusion
- all other unexpected adverse events requiring the termination of eplerenone treatment

2) present with an indication for treatment that is contraindicated in association with eplerenone (trimethropim, digoxin …)

5.3.2 Methods and timetable for the collection of data (criteria for termination)

See Table 1 and Table 2 section 4.2.

5.3.3 Methods of replacement of subjects, if the case arises

Not applicable

5.3.4 Methods of follow-up of the subjects

See section 4.2.

6. TREATMENT ADMINISTERED TO SUBJECTS TAKING PART IN THE STUDY

6.1. Description of treatment(s) necessary for the realisation of the study

The main treatment for this study is eplerenone (see section 4.4.2).

6.2. Associated medicines and treatments allowed or forbidden by the protocol, including rescue medications

In cases of the development an adverse event, other treatments could be necessary for the realisation of this study:
- sodium polystyrene sulfonate (Kayexalate®): this is a powder for suspension administered orally or rectally and sold in bottles of 454 g with a measuring spoon; it is administered daily to treated hyperkalaemia

- sodium bicarbonate®: this consists of an extemporaneous capsule preparation containing 500 mg of sodium bicarbonate; it is administered daily to treat metabolic acidosis.

These treatments will be given to the patients by the promotor. The pharmacy associated with this study is the pharmacy of the CHU, Reims (Hôpital Robert Debré); the pharmacist responsible for the study is Mr Philippe Benoit.
During this study, no medicine or treatment (except for those listed in the exclusion criteria) is forbidden but they should be mentioned as quickly as possible to the investigators and noted in the observation folder.

6.3. Methods of follow-up and compliance with treatment

The team of the central pharmacy of the CHU, Reims, will recover all empty packaging of eplerenone, of Kayexalate® and of sodium bicarbonate® at the end of each step in order to follow-up compliance with treatment. Treatments not administered will also be recovered.

6.4. Conditions for the storage of the experimental drugs

The experimental drugs will be stored by the central pharmacy before their delivery. Once delivered, the experimental medicines should be stored at room temperature, in the shade.

7. EVALUATION OF EFFICACY

7.1. Description of the parameters for the evaluation of efficacy (evaluation criteria)

Not applicable

7.2. Methods and expected timetable for the measurement, collection and analysis of parameters for the evaluation of efficacy

Not applicable

8. EVALUATION OF SAFETY

8.1. Description of the parameters for the evaluation of safety

See section 4.2.

8.2. Methods and expected timetable for the measurement, collection and analysis of parameters for the evaluation of safety

During each follow-up consultation (which should take place every 28 days), the investigator will assess the development of adverse events and will evaluate the safety of eplerenone in the patients taking part in the study.

Prevention of the adverse events of eplerenone
At inclusion, the investigator will inform the patient about the risks of hyperkalaemia and the risks linked to hyperkalaemia. In addition, they will give the patient a letter explaining the optimum diet during chronic renal failure, limiting the supply of potassium. They will give the patient, at the inclusion consultation, a box of KAYEXALATE® to have available during the study. A prescription leaflet, carrying a clear explanation of how the medicine should be used, will be given to the patient.

Procedure to follow in the case of an adverse event

1) Hyperkalaemia

In the case of mild hyperkalaemia (kalaemia ≥5 mmol/L but <5.5 mmol/L), the investigator will contact the patient and remind them about the hygieno-dietary rules concerning a low potassium diet.
In the case of significant hyperkalaemia (kalaemia ≥5.5mmol/L but <6 mmol/L) detected for the first time, the investigator will contact the patient and remind them about the hygieno-dietary rules concerning a low potassium diet and tell them to take 1 spoon-measure of KAYEXALATE® the same day, as well as measuring their kalaemia in the following 24 hours.
In the case of significant hyperkalaemia (kalaemia ≥5.5 mmol/L but <6 mmol/L) noted at the time of measurement after taking 1 spoon-measure of KAYEXALATE®, the investigator will contact the patient and remind them about the hygieno-dietary rules concerning a low potassium diet and tell them to take 2 spoon-measures of KAYEXALATE® the same day as well as measuring their kalaemia in the following 24 hours.
In the case of hyperkalaemia ≥6 mmol/L, or ≥5.5 mmol/L despite 2 spoon-measures of KAYEXALATE®, treatment with eplerenone will be stopped, treatment with KAYEXALATE® strengthened (3 spoon-measures) and the patient seen in an emergency consultation the same day with an ECG (electrocardiogram).

2) Metabolic acidosis

In the case of mild metabolic acidosis (alkaline reserve ≤20 mmol/L but >15 mmol/L), the investigator will tell the patient to take 6 capsules of sodium bicarbonate 500 mg® the same day as well as measuring their alkaline reserve at the time of the next assessment.
In the case of significant metabolic acidosis (alkaline reserve ≤15 mmol/L) noted during the measurement after taking 6 capsules of sodium bicarbonate 500 mg® or spontaneously, treatment with eplerenone will be stopped, treatment with sodium bicarbonate 500 mg® doubled (12 capsules/day) and the alkaline reserve measured the following week.

3) Other

Concerning the development of an adverse event not anticipated in the study protocol, the patient will be informed at the time of inclusion in the study that they should alert the investigator as quickly as possible about any unusual event. To do this, they can contact the Nephrology Service of the CHU, Reims, in which a physician is contactable 24h/24 and 7D/7. This event should be noted as early as possible in the observation file.
In the case of hospitalisation or the initiation of a new treatment during the study, the patient will be informed that they should notify the investigator as quickly as possible.
Renal transplant patients followed in the Nephrology Service of the CHU, Reims, are all informed that they should contact the service if any new medical event should occur. They can attend without an appointment during the opening hours of the NSDH or contact a nephrologist 24h/24 and 7D/7.

8.3. Procedures for the recording and notification of adverse events

At each consultation, the investigator will question the patient about the appearance of any adverse events and will note them in the observation file.
Arterial pressure will be measured at each consultation for the assessment of arterial hypotension or orthostatic hypotension. These values will be noted in the observation file.
Finally, any biological adverse events (kalaemia, alkaline reserve, creatininaemia) will be noted prospectively by the Clinical Research Assistant (CRA) in the observation file, after having ensured that the investigator is aware of them.
In accordance with the law of 9th August 2004 of the Public Health Code, transposing the European Directive into French law, the declaration of adverse events will comply with a procedure

established at the start of the study. For this study, these declarations will comply with internal Standardised Operating Procedure SOP N°9 of the CHU, Reims, relating to the declaration of adverse events and to safety during clinical trials of medicines for human use conducted in France, of which the CHU, Reims, is the promotor.
This specifies that the Pharmacovigilance Unit (PU) of the CHU, Reims, should be notified about any adverse events by Fax (to: 03.26.83.23.79) as quickly as possible using a declaration form (see **Figure 2**).

Réservé CRPV Reims : Cas RE.....................

**Promoteur :** **CHU DE REIMS**	**DECLARATION D'EVENEMENT INDESIRABLE GRAVE (EIG)** Cette fiche est à faxer dûment complétée dans les 24 heures ouvrées A l'Unité de Pharmacovigilance (UPV) du CHU de Reims au **03.26.83.23.79**

Chaque EIG doit être décrit sur une fiche séparée et numérotée **Fiche … sur …** **pour le patient n°** **1/3**

A- IDENTIFICATION DU PROTOCOLE
Référence de l'essai : n° — n° EudraCT :
Produit à l'essai : — Titre de l'essai :

Coordonnées investigateur :
Nom : — Fonction :
Service : — Téléphone :

Date de transmission : /_/_/ /_/_/ /_/_/_/_/ (JJ MM AAAA)
☐ Notification initiale
☐ Suivi n°.........

B- INFORMATIONS SUR LE PATIENT

N° de patient : .... Bras (si applicable) :

Sexe : ☐ Homme ☐ Femme ☐ Femme enceinte *(Date des dernières règles : ....)*

Nom (3 premières lettres) : / / / / Prénom (Initiale) : / / Date de naissance : / / / / / / / / / / /
Poids (kg) : .... Taille (cm) : ....

Antécédents pertinents :
- ....
- ....
- ....
- ....
- ....

Pathologies concomitantes :
- .... depuis le : ....
- .... depuis le : ....
- .... depuis le : ....
- .... depuis le : ....
- .... depuis le : ....

C- INFORMATIONS SUR LE(S) MEDICAMENT(S) A L'ESSAI

	DCI ou nom commercial	Voie d'administration	Dosage	Posologie	Date de début	Heure de début (si durée <24h)	Date de fin	Heure de fin (si durée <24h)
1								
2								
3								

Levée d'aveugle ☐ Oui ☐ Non ☐ Non applicable
Si oui, date de levée d'aveugle : /_/_/ /_/_/ /_/_/_/_/
Résultat : le traitement reçu est le ....

CRPV de Reims – Avenue du Général Koënig - Hôpital Robert Debré - 51092 Reims Cedex

Réservé CRPV Reims : Cas RE.....................

Chaque EIG doit être décrit sur une fiche séparée et numérotée **Fiche … sur …** **pour le patient n°** **2/3**

D- INFORMATIONS SUR LE(S) MEDICAMENT(S) ASSOCIES *(à l'exclusion de ceux utilisés pour traiter l'événement)*

	DCI ou nom commercial	Voie d'administration	Dosage	Posologie	Date de début	Heure de début (si durée <24h)	Encore en cours	Date de fin	Heure de fin (si durée <24h)	Indication
1										
2										
3										

E- EVENEMENT INDESIRABLE GRAVE
Désignation : ....

☐ **Evénement grave car :**
- ☐ Fatal
- ☐ Susceptible de mettre la vie en danger
- ☐ Provoque une hospitalisation* / prolongation d'hospitalisation existante
  Date d'admission : .... Date de sortie : ....
  **Exceptées les hospitalisations dont la durée et le but sont planifiés dans le protocole*
- ☐ Incapacité / invalidité persistante ou importante
- ☐ Grossesse / Anomalie congénitale à la naissance
- ☐ Intervention nécessaire pour éviter l'une des évolutions ci-dessus

☐ **Autre événement clinique / Laboratoire potentiellement grave** (médicalement significatif comme décrit dans le protocole)

Période de survenue de l'EIG :
- ☐ Pendant la phase de sélection
- ☐ Pendant la phase d'administration du produit à l'essai
- ☐ Après l'arrêt de l'administration du traitement à l'essai (phase de suivi)

Date de début de l'EIG : /_/_/ /_/_/ /_/_/_/_/ et heure de survenue (si EIG <24h) : ....

Description (Diagnostic : décrire la chronologie de l'événement. Joindre les compte-rendus anonymisés d'hospitalisation, d'examens et/ou résultats de laboratoire si nécessaire)
____
____
____
____
Mesures thérapeutiques prises : ☐ Non ☐ Oui, si oui description : ______________________________

CRPV de Reims – Avenue du Général Koënig - Hôpital Robert Debré - 51092 Reims Cedex

Réservé CRPV Reims : Cas RE.....................

Chaque EIG doit être décrit sur une fiche séparée et numérotée **Fiche** ... **sur** ... **pour le patient n°** **3/3**

E- EVENEMENT INDESIRABLE GRAVE

Disparition de l'EIG après arrêt du ou des traitement(s) à l'essai : ☐ Oui (n° 1 - 2 – 3)* ☐ Non ☐ Sans objet
Réapparition de l'événement après réintroduction du ou des traitement(s) à l'essai : ☐ Oui (n° 1 – 2 – 3)* ☐ Non ☐ Sans objet

Evolution de l'événement : ☐ Guérison sans séquelle
☐ Guérison avec séquelle
☐ Sujet non encore rétabli avec évolution par rapport à l'état initial : ☐ Améliorée ☐ Inchangée ☐ Aggravée

☐ Décès : ☐ En rapport avec l'EIG ☐ Sans rapport avec l'EIG
Date du décès /_/_/ /_/_/ /_/_/_/_/
Autopsie : ☐ Oui (joindre le rapport d'autopsie si disponible) ☐ Non

F- MESURES PRISES SUITE A L'EIG

Produit(s) à l'essai
- ☐ Traitement poursuivi sans changement
- ☐ Modification de dose(s) : Nouvelle posologie : .... depuis le ....
- ☐ Arrêt temporaire du traitement à l'essai (n° 1 – 2 – 3)* du .... au ....
- ☐ Arrêt définitif du traitement à l'essai (n° 1 – 2 – 3)* : Date de fin : ....
- ☐ Non applicable

L'EIG a conduit à un arrêt des médicaments associés : ☐ Non ☐ Oui, si oui *numéro 1 – 2 – 3* : ☐ Arrêt temporaire
☐ Arrêt définitif

G- EVALUATION DU LIEN DE CAUSALITE

Selon l'investigateur l'EIG semble plutôt lié :
☐ au(x) traitement(s) à l'essai (n°1 – 2 – 3)*
☐ autre cause : ☐ traitement associé (n°1 – 2 – 3)*
☐ maladie intercurrente
☐ progression de la maladie
☐ autre raison : ....
☐ information insuffisante

CADRE RESERVE AU CRPV

Selon le promoteur l'EIG semble plutôt lié :
☐ au(x) traitement(s) à l'essai (n°1 – 2 – 3)*
☐ autre cause : ☐ traitement associé (n°1 – 2 – 3)*
☐ maladie intercurrente
☐ progression de la maladie
☐ autre raison : ....
☐ information insuffisante

Si l'EIG est lié au médicament évalué :
☐ l'événement indésirable est attendu
☐ l'événement indésirable est inattendu

**Entourer le numéro de traitement correspondant*

Nom de l'investigateur .... **Date :** .... **Signature :**

CRPV de Reims – Avenue du Général Koënig - Hôpital Robert Debré - 51092 Reims Cedex

Figure 2. Form for the declaration of an adverse event

8.4. Methods and duration of follow-up of subjects following the development of an adverse event

See section 8.2.

9. STATISTICS

9.1. Description of the statistical methods planned, including the timetable for any intermediate analyses if applicable

Global analysis
The analysis of the primary evaluation criterion will be carried out by a detailed breakdown as soon as the development of an adverse event(s) necessitates the termination of eplerenone treatment (see section 4.2). This will be a descriptive analysis.
The analysis of the secondary criteria will be descriptive and comparative between before and under eplerenone treatment.
Discontinuous variables will be expressed as percentages and continuous variables as the mean ± standard deviation.

Intermediate analyses
Not applicable.

9.2. Number of subjects to include in the study with statistical justification and theoretical number of patients expected in each place of study, if applicable

The anticipated number of subjects to include in the study is 31 patients.
See section 5.3 for the procedure for termination of the study
See section 9.3 for the justification of number of patients to include
See section 9.1 for the methods of analysis of the data

9.3. Size of the sample / population

9.3.1 Calculation and/or justification of the number of subjects required

9.3.1.1. Baseline hypothese(s) for this calculation:

According to data in the literature, the probabilities of hyperkalaemia developing during 8 weeks of eplerenone treatment are <7% with the 25 mg dose and <10% with the 50 mg dose (Gonzalez Monte *et al.*, 2010, Pitt *et al.*, 2003, Pitt *et al.*, 1999, Zannad *et al.*). We posed the alternative hypothesis that it is not useful to continue the study if the risk of hyperkalaemia is >15% with the 25 mg dose and 20% with the 50 mg dose.
The power for detecting an adverse event requiring the termination of treatment was fixed at 90%.
Under these hypotheses, it is possible to carry out a study in several steps by adapting the 2-step scheme proposed by Simon (Simon, 1989) in the following way:
First step: 14 patients will be included at the dose of 25 mg and followed for 8 weeks: if ≥4 patients experience an adverse event requiring the termination of treatment, the study will be stopped. In the opposite case, 17 additional patients will be included at the same dose for 8 weeks. If ≥5 patients out of the 31 have a limiting toxicity, the alternative hypothesis can be rejected and the study will be stopped; in the opposite case, the dose will be increased to 50 mg for 31 patients with a follow-up at 6 months. If at the end of this follow-up, ≤4 patients have a limiting toxicity with the 50 mg dose, the alternative hypothesis will be rejected and continuation of the study with eplerenone will be possible.
In these conditions, the probability of demonstrating a limiting toxicity greater than the fixed norms is 90.0%, the risk of stopping the study wrongly is 27.6%.

9.3.1.2. Level of reference of the evaluation criterion and its variability

The investigators anticipate a maximum of 15% adverse events requiring the stopping of eplerenone treatment in this population at a dose of 25 mg/day and 20% at a dose of 50mg/day.
They do not foresee any variability for this evaluation criterion.

9.3.1.3. Statistical risks chosen (α, β)

The α risk chosen is 27.6%.
The β risk chosen is 10%.

9.3.1.4. Theoretical calculation of the number of subjects per group

A single group will be necessary in this study. The size of this group is calculated as 31 patients.

9.3.1.5. Estimation of the number of subjects to include (from the theoretical calculation and taking into account the estimated percentage of subjects lost to follow-up or not evaluable)

The investigators do not anticipate having any patients lost to follow-up. This population is already well known by the investigation centre because it consists of patients already followed for at least 2 years by this centre at the date of inclusion. It consists of patients who are necessarily compliant since the immunosuppressive treatments should be taken regularly at strict times. These patients are used to being monitored closely by this centre and to undergoing examinations necessary to this care.

9.3.2 Method of sampling

The patients will be selected from the cohort of renal transplant patients followed-up by the Nephrology Service of the CHU, Reims.
They will be chosen from among those who agree to participate in the study and who satisfy the inclusion and exclusion criteria.

9.3.3 Methods of random sampling

Not applicable

9.4. Statistical methods for the analysis of the evaluation criteria

9.4.1 For the primary criterion

See section 9.1

9.4.2 For the secondary criteria

See section 9.1

9.4.3 Specify the threshold of statistical significance used

Not applicable

9.5. Treatment of missing data

The investigators do not anticipate having any missing data taking into account the characteristics of the study population as described in section 9.3.1.5.
Furthermore, the follow-up of renal transplant patients has been assisted, since 2005, by computer software (Hémadialyse©) in which the previous medical history, medical events and consultation and hospitalisation reports of these patients are recorded.

9.6. Management of data for the patients

See Declaration CNIL: Chapter 9 of the law of 6th January 1978 modified by the law of 6th August 2004: « obtain the favourable opinion of the Consultative Committee on the treatment of information in the area of health ».

The data will be collected prospectively throughout the study and noted in the observation files as the study progresses. The observation files will be stored in a locked cupboard in a locked office. Access to this office will be restricted to the investigators and CRAs.
The constitution of the database as well as its management will be carried out by CRICAM (Centre for Research, Clinical Investigation and Methological Assistance) of the CHU, Reims.

10. RIGHT OF ACCESS TO THE DATA AND SOURCE DOCUMENTS

In accordance with current laws and regulations, notably articles L.1121-3 and R.5121-13 of the CSP, individuals having direct access (investigators, promotor CRA, quality control CRA, non-medical researchers participating in the study or all other persons duly authorised by the promotor ….) are subject to professional confidentiality. Furthermore, these persons will take all precautions necessary to assure the confidentiality of information relating to experimental medicines (if the cases arises), to trials, to individuals who take part in them and notably, regarding their identity as well as the results.
Comment: The data collected by these individuals during quality control or audits will be anonymised.

11. QUALITY CONTROL AND ASSURANCE

In order to assure the quality and smooth progress of the study, the principal investigator will set up a Steering Committee. This committee will include at a minimum, all the investigators in the study but may also include the methodologists and any individual that the investigator feels useful to the smooth progress of the study or necessary to guarantee the quality of the study. The list will be defined before the start of the study.
It will meet at least 3 times during the study:

- before the start of the study in order to organise the progression of the study
- in the middle of the study in order to assure the smooth progress of the study
- at the end of the study in order to organise the treatment of the data

If it is considered necessary to the quality of the study, the principal investigator could convene the Steering Committee more often.
Each meeting will give rise to the drafting of a detailed report.

Quality control of the data

A control of the quality of the data will be carried out by random sampling of 10% of the files by a promotor CRA.
See Annexe 1 of SOP N°10 « Quality assurance – Monitoring » of the CHU, Reims.

12. ETHICAL CONSIDERATIONS

12.1. Law for Biomedical Research and Bioethics

Promotor: CHU, Reims
Principal investigator: Pr. Philippe RIEU
Committee for the Protection of Persons: EST III

12.2. Benefit/risk ratio

This will be a study of the safety of use of a medicine which already has MA but is outside the title of the MA.
The risks for the patients taking part in this study are essentially the development of hyperkalaemia and metabolic acidosis. Taking into account the very close monitoring during the study and measures for the rapid management of these adverse events, the risks are controlled to a maximum. Moreover, these adverse events are rapidly reversible on stopping eplerenone.
The expected benefits for the patients taking part in the study are small. The time to the effect of eplerenone on renal function is much longer than the duration of this study. In this study, we wish to evaluate the risk of using this molecule before demonstrating its efficacy more long-term. It is at that moment that a benefit will be expected in the population taking part in the study.

12.3. Information letter and consent form for the subjects

Information letter and consent form for subjects involved in studies governed by the Law for Biomedical Research:

INFORMATION LETTER
FOR PATIENTS
FOR PARTICIPATION IN BIOMEDICAL RESEARCH

Title of the study: STUDY OF THE SAFETY OF EPLERENONE IN RENAL TRANSPLANT PATIENTS TREATED WITH CYCLOSPORINE
EPLErenone in CsA-Treated patients (EPLECsAT): safety

Dear sir, madam,

We offer you the opportunity to take part in a clinical study.
The coordinator of this study, for which the CHU, Reims, is the promotor, is Professor Rieu from the Nephrology Service.

Participation in this study is completely voluntary and will not cost you anything. In accordance with the law, all costs linked to the study will paid by the study promotor. If you do not understand some of the information, do not hesitate to ask your doctor for an explanation. To participate in this study, you must be affiliated to a social security regime.

The aim of this study is to evaluate the tolerance of eplerenone treatment in renal transplant patients treated with cyclosporine. This use of eplerenone is not one of the authorisations obtained for this treatment, this indication is therefore outside its Marketing Authorisation (MA). Nevertheless, we think that eplerenone could prevent some of the renal side-effects of cyclosporine. This medicine could also slow down the loss of renal graft function. Before being able to demonstrate this hypothesis in a large clinical trial, it is necessary to ensure that eplerenone can be used safely in renal transplant patients treated with cyclosporine. As with all effective treatments, the use of eplerenone can result in undesirable side-effects or complications which, in the case of renal failure and associated treatment with cyclosporine, may be: hyperkalaemia (increase in potassium in the blood), metabolic acidosis (acidification of the blood) and a decrease in blood pressure. The aim of the study proposed is to investigate the tolerance of eplerenone treatment and, in particular, to evaluate the risk of hyperkalaemia.
You can discuss this list of possible side-effects with your doctor in order to better make the distinction between very rare events and those that may occur more frequently. Evidently, your doctor will be attentive to all problems of tolerance of treatment, of aggravation of clinical symptoms requiring another rapid treatment, or any problem concerning you during follow-up which could justify your withdrawal from the study.

We plan to recruit 31 patients from the CHU, Reims, for participation in this study, which will last for 6 months. The duration of your participation will be from 16 to 24 weeks divided into 2 or 3 periods of 8 weeks.

The first visit will serve to verify that you fulfil the criteria for inclusion in the study. Before the treatment can start, a clinical examination as well as blood and urine investigations will be carried out. You will be told the results of these examinations directly by the investigator or by a doctor of your choice. It is important that you respond to your doctor's questions, with the greatest accuracy on your symptoms, your illnesses and your treatments, past or ongoing. These examinations will help to verify that you fulfil the necessary criteria for inclusion in the study.

Treatment with eplerenone will be started at a low dose (25 mg/day) during the first period. In the absence of any side-effect(s), the dose will be increased to 50 mg/day during the second period. Surveillance of kalaemia will take place via a blood sample taken 2 days after the start of each period and then once a week. The blood sample can be taken in an agreed laboratory close to your home or in hospital. The results will immediately be communicated to the investigators.

The effect of treatment will also be assessed by a consultation every month except at the start of treatment and during the changes in dose when an additional consultation will take place at 14 days, an analysis of urine monthly, and a Doppler-echography of the graft at the start and end of the study.

The expected benefits of this study to you are small but the results collected could possibly serve to carry out a larger study which could help in the treatment of other patients in the future.

The constraints for you will be the monthly follow-up consultations, the regular blood sampling (every week and 48 hours after each change of dose) as well as a low potassium diet. Furthermore, you should be contactable by telephone during the 8 hours that follow each blood sample in order to be informed of the results and any modifications in treatment.

You can refuse to participate in this study or withdraw your consent at any time during the study and no longer take part (without having to give a reason) without this influencing your relationship with your doctor.

We should point out that during the time of your participation in this study, and for 1 month after the end of your participation, we ask you to not take part in another therapeutic trial.

In accordance with French law, the Committee for the Protection of Persons (CPP) Est III has studied this research project and has granted its approval for the study to be carried out, on the **xx/xx/xxxx**. The Competent Authority has also granted its approval, on the **xx/xx/xxxx**.

The promotor of the study « CHU, Reims, Avenue Cognacq Jay, 51092 Reims » has taken out an insurance policy «policy number: **xxxxx** » at the request of the company: SHAM to cover the risks linked to this investigation.

All information concerning you and collected during this study will be treated confidentially.

Only those responsible for the study and possibly the Health Authorities can have access to this data. With the exception of these individuals – who will treat the information with strict medical confidence - your anonymity will be preserved. Publication of the results of the study will not include any individual results.

The data collected during this study will be the object of computer treatment by the promotor. These data will be identified by a code number and the first three letters of your surname and first name. Consisting of nominative data, you will have the right to access and to rectify the data concerning you at any time with those responsible for the study. Concerning information of a medical nature, this right is exercised through the intermediary Doctor ……………………… in accordance with law 78-17 of 6th January 1978 relating to the Data Protection Act, modified by law n°94-548 of 1st July 1994, concerning the

treatment of nominative data for health-related research. The project has been approved by the National Commission for Computing and Liberties (CNIL) on **xx/xx/xxxx**.

In accordance with article L1122-1 of the Public Health Code (law of March 2002 concerning the rights of patients), the overall results of the study will be communicated to you if you wish.

You can also access, directly or through the intermediary of a doctor of your choice, all of your medical data subject to the provisions of article L1111-7 of the Public Health Code. These rights can be exercised through the doctor who will monitor you in the study and who knows your identity.

The results of this study may be the object of communications at medical conferences and/or be published in a scientific journal.

We thank you for taking the time to read this information letter. If you would like to take part in this study, please sign the attached consent form.

CONSENT FORM
FOR THE PARTICIPATION IN BIOMEDICAL RESEARCH

TITLE OF STUDY: STUDY OF THE SAFETY OF EPLERENONE IN RENAL TRANSPLANT PATIENTS TREATED WITH CYCLOSPORINE
EPLErenone in CsA-Treated patients (EPLECsAT): safety

I, undersigned ……………………………………………………, accept to participate in the study of EPLErenone in CsA-Treated patients (**EPLECsAT**): safety

The objectives and methods of the study organised by the CHU, Reims, have been clearly explained to me by Dr………………...
He/she has explained that I am free to accept or refuse. This will not change our relationship regarding my treatment.

I have read the information letter that was given to me.
In order to clarify my decision, I have received, from the investigator, an explanation of the nature, expected benefits, constraints and anticipated risks of the study, the methods of medical management anticipated at the end of the study (in the case of premature withdrawal, in the case of exclusion from the study) and I have been told about the duration and progression of the study.

I have understood this information and he/she has left me the time and leisure to ask all the questions that I wanted.

I will be informed, during the course of the study, of any new factor that may change my decision to participate in the study.

I understand that I should not take any new treatment without first speaking to my doctor.
I understand that I should not participate in another study during this study and for 1 month after the end of the study.

I accept that the documents from my file that are relevant to the study can be accessed by those responsible for the study and possibly by the health authorities. With the exception of these individuals, who will treat the information in the strictest confidence, my anonymity will be preserved. Moreover, I authorise the promotor to keep a copy of the present consent in a sealed envelope guaranteeing complete confidentiality.

I accept that the nominative data concerning me and collected during this study may be the object of automated treatment by the study organisers. I can exercise my right to access and rectify the data according to Dr ……………..
If any questions or problems arise during the study, I can contact the doctor in charge of this study on [telephone number]…………………………………………

I have been informed that this study will be conducted in accordance with current French laws and that it has been granted approval by the Committee for the Protection of Persons (CPP), Nancy, on ……………………as well as authorisation from the Competent Authority on ……………

I understand that my participation in the study is entirely voluntary.

I am free to accept or to refuse to participate, and I am free to stop my participation at any time during the study. This will not affect the quality of care that will I be given.

My consent does not discharge the organisers of this study from their responsibilities. I conserve all my rights guaranteed by law.

After having discussed and obtained answers to all my questions, I freely and willingly accept to participate in the study that has been proposed to me clearly in this document.

I accept that the data recorded during this study may be the object of computerised processing by the promotor or on his behalf. I have noted that the right of access established by the Data Protection Act can be exercised at any time through Dr ………………………………. I can exercise my right to rectify the data according to the same doctor.

In the case of publication of the results in a medical or scientific journal, my identity will not be revealed.

I certify that I have been given an example of the information letter and give my clear and written consent.

✍ *To be completed by the patient*

Patient :
Surname/ first name, in capital letters:
Date:
Signature:

✍ *To be completed by the investigator*

Investigator:
Surname / first name, in capital letters:
Date:
Signature:

Original for the investigator, 1 copy for the patient and 1 copy for the promotor

13. TREATMENT OF THE DATA AND STORAGE OF DOCUMENTS AND DATA RELATING TO THE STUDY

13.1. Recording, collection and treatment of data

The recording of data will be carried out by the investigator at the time of each consultation.
The collection of clinical data will be carried out by the promotor CRA.
The recording and collection of biological data will be carried out by the promotor CRA.
The treatment of data will be carried out by the biostatistics group of CRICAM of the CHU, Reims.

13.2. Collection of data

All data will be centralised by the coordinator who will organise data collection in the computer software of CRICAM after having verified the notes in the observation file.
Manual centralised double entry of the data will be carried out systematically. An automated comparison of the data will be carried out and all incomplete or aberrant data will be the object of consultation of the paper file.
A control of the quality of the data will be carried out randomly on 10% of the files by a promotor CRA.

13.3. Logistics of treatment of data

The analysis of the data will be carried out by CRICAM at the CHU, Reims.

13.4. Storage of documents and data

See section 9.6.
As the study relates to the Law for Biomedical Research and conforms to current laws and regulations, the documents will be stored by the promotor and by the principal investigator for a period of 15 years from the date of the end of the study.

14. FUNDING AND INSURANCE

An evaluation of the budget necessary for this study to be carried out has been performed. The total amount is 34 471.00 € (incl. taxes). The amount relating to eplerenone itself is 8 512.00 € (incl. taxes): in the case of participation of the pharmaceutical company in this study, eplerenone will be provided by the manufacturer.
A part of this budget is the object of funding by the AOL 2011 of the CHU, Reims.
The remaining funding is the object of co-funding by Team 1 of INSERM U872, directed by Dr JAISSER,
Insurance will specifically be taken out by the promotor, the CHU, Reims, for this study.

15. RULES RELATING TO PUBLICATION

Any publication originating from this study will describe the overall results and their analysis.
The principal investigator will systematically be the last author of any publication originating from this study. He will give his support for any publication derived from this study.
The contents and order of the other authors will be discussed among the groups participating in the study.

16. LIST OF ANNEXES

16.1. Annexe 1: List of investigators

Principal investigator:
Pr. RIEU
Head of Nephrology Service - Arterial Hypertension - Haemodialysis – Transplantation, CHU, Reims.

Co- investigators
Dr TOUPANCE
Nephrology Service - Arterial Hypertension - Haemodialysis – Transplantation, CHU, Reims.

Dr LAVAUD
Nephrology Service - Arterial Hypertension - Haemodialysis – Transplantation, CHU, Reims.

16.2. Annexe 2: List of scientific collaborators, if applicable

Jean-Philippe BERTOCCHIO (Intern DES Nephrology)
Nephrology Service - Arterial Hypertension - Haemodialysis – Transplantation, CHU, Reims.

Dr Frédéric JAISSER
Director of Research, INSERM
INSERM U872 Equipe 1

Pr. Pierre NAZEYROLLAS
Delegated Physician,
Centre for Research and Clinical Investigation

17. SIGNATURE PAGES

Promotor: CHU, Reims

Signature

Investigator/research coordinator:
Pr. Philippe RIEU
Research coordinator

Signature

External establishments involved (CHU, Region, Inter-region)

INSERM U872, Equipe 1 (co-funding)
Directed by Dr JAISSER
Centre de Recherche des Cordeliers
15 rue de l'Ecole de Médecine
75006 Paris CEDEX
France.
